# Cognitive profiling and proteomic analysis of the modafinil analogue S-CE-123 in experienced aged rats

**DOI:** 10.1038/s41598-021-03372-y

**Published:** 2021-12-14

**Authors:** István Gyertyán, Jana Lubec, Alíz Judit Ernyey, Christopher Gerner, Ferenc Kassai, Predrag Kalaba, Kata Kozma, Iva Cobankovic, Gábor Brenner, Judith Wackerlig, Eva Franschitz, Ernst Urban, Thierry Langer, Jovana Malikovic, Gert Lubec

**Affiliations:** 1 Department of Pharmacology and Pharmacotherapy, Semmelweis University, Nagyvárad tér 4., Budapest, 1089 Hungary; 2 Programme of Proteomics, Department of Neuroproteomics, Paracelsus Private Medical University, Strubergasse 21, 5020 Salzburg, Austria; 3 Department of Analytical Chemistry, University of Vienna, Vienna, Austria; 4 Department of Pharmaceutical Chemistry, University of Vienna, Vienna, Austria; 5 Core Unit of Biomedical Research, Division of Laboratory Animal Science and Genetics, Medical University of Vienna, Himberg, Austria

**Keywords:** Pharmacology, Dementia, Learning and memory, Proteomics

## Abstract

The lack of novel cognitive enhancer drugs in the clinic highlights the prediction problems of animal assays. The objective of the current study was to test a putative cognitive enhancer in a rodent cognitive test system with improved translational validity and clinical predictivity. Cognitive profiling was complemented with post mortem proteomic analysis. Twenty-seven male Lister Hooded rats (26 months old) having learned several cognitive tasks were subchronically treated with S-CE-123 (CE-123) in a randomized blind experiment. Rats were sacrificed after the last behavioural procedure and plasma and brains were collected. A label-free quantification approach was used to characterize proteomic changes in the synaptosomal fraction of the prefrontal cortex. CE-123 markedly enhanced motivation which resulted in superior performance in a new-to-learn operant discrimination task and in a cooperation assay of social cognition, and mildly increased impulsivity. The compound did not affect attention, spatial and motor learning. Proteomic quantification revealed 182 protein groups significantly different between treatment groups containing several proteins associated with aging and neurodegeneration. Bioinformatic analysis showed the most relevant clusters delineating synaptic vesicle recycling, synapse organisation and antioxidant activity. The cognitive profile of CE-123 mapped by the test system resembles that of modafinil in the clinic showing the translational validity of the test system. The findings of modulated synaptic systems are paralleling behavioral results and are in line with previous evidence for the role of altered synaptosomal protein groups in mechanisms of cognitive function.

## Introduction

Modafinil is approved as an eugeroic, however its cognitive effects have long been studied in healthy volunteers^1–3^ as well as in sleep-deprived individuals^2,4^ and attention deficit hyperactivity disorder (ADHD) patients^5^. Although modafinil is a known atypical dopamine transporter (DAT) inhibitor, it is not only targeting DAT but also the serotonin and the norepinephrine transporter. This formed the rationale to synthetize more potent compounds with higher specificity. Indeed, CE-123 was superior to racemic and R-modafinil, the lead compound, in affinity, specificity and dopamine reuptake inhibition^6^. The efficacy of CE-123, both racemic and its S-enantiomer on cognitive and executive functions was shown in several studies and in several cognitive assays^6–10^. However, the compound has not been tested with multiple dosing and in aged animals yet.

A complex rat cognitive test system designed to improve the predictivity and translational value of animal studies was used to investigate procognitive effect of CE-123. The theoretical basis of the system was described in^11^ and^12^. Briefly, rats are taught several learning tasks representing models of human cognitive domains studied in the clinical trials. Thereby a population with “widespread knowledge” is brought about. Then this population is exposed to a certain intervention impairing their cognitive performance, thus a “patient population” is created. This “patient population” serves as subjects of a clinical trial-like study with a putative cognitive enhancer. In the current study rats previously having acquired the 5-choice serial reaction time task (5-CSRTT, model of sustained attention), the Morris water-maze task (model of spatial learning), a cooperation task^13^ (model of social learning) and the so-called pot jumping task^14^ (model of motor learning), took part and aging itself was the impairing intervention coupled with increasing task difficulty in the attentional task. In addition, rats had to learn a completely new operant discrimination task. Testing the proposed procognitive effects of CE-123 was carried out in a randomized, blinded study with chronic treatment. Previous results obtained in the model^15,16^ showed that experienced animals are quite resistant to the impairing interventions. This finding adds an important, and so far overlooked aspect to the human relevance of the system. We assume that long-term studies in animals with considerable learning experience are more predictive than acute studies in naïve or freshly trained animals. Therefore, the aim of the study was on one hand the cognitive profiling of CE-123 in aged experienced animals with subsequent proteomic analysis, and on the other hand showing and proposing the utility of the test system described above.

## Methods and materials

### Animals

Twenty-seven male Lister hooded rats (Charles River, Germany), 26 months of age at the start of the study were used. They had a previous history of acquiring and routinely performing several learning tasks. The animals' housing conditions and learning history are reported in detail in the Supplemental Information (SI) file. Housing and all procedures carried out on animals were authorized by the regional animal health authority in Hungary (Pest County Government Office, resolution number PEI/001/3572-4/2014) and conformed to the Hungarian welfare legislation, the EU 63/2010 Directive, and ARRIVE guidelines.

### Learning paradigms

Detailed methodical descriptions can be found in the Supplemental Information file.

#### 5-choice serial reaction time test (5-CSRTT)

The operant chamber was equipped with five nose-poke modules. Animals were trained to nose-poke into a randomly chosen hole marked for 1 s to get a food pellet as a reward (correct response). The animal made an incorrect response if nose-poked into one of the non-signalled holes, an omission if it did not respond to the stimulus during its duration plus a 5 s long hold period, and a premature response if nose-poked into any of the holes during the 5 s long inter-trial interval. Rats learned the task before their first year of age (*see* SI for details), and then they participated in regular maintenance training until the start of this study. Here, task difficulty was increased by reducing the stimulus duration to 0.25 s. The outcome parameters were the following: number of initiated trials, number of rewards obtained (equals number of correct responses), % correct response ratio (correct responses/(total trials-premature responses) × 100), % omission ratio (omissions/(total trials-premature responses) × 100), % premature response ratio (premature responses/total trials × 100).

#### Morris water-maze (MWM)

The task of the animals was to find a hidden platform in a circular tank of 190 cm diameter filled with water. The platform was 1 cm under the water surface in the south east quadrant and rats were placed in the water at either of the north, east, south or west edge of the pool. On the wall of the experimental room extra-maze cues were placed to facilitate orientation during swimming. Animals were given 180 s to escape to the hidden platform and their movement was recorded with a video tracking software. Animals were acquainted with the MWM paradigm before the age of 7 months. From the age of 20 months, they underwent maintenance training sessions with varying platform location rotating among the four quadrants of the pool. The last session (platform located in the north-east) served as the baseline measurement. However, as the animals were getting older, swimming for longer intervals exhausted them; therefore, the trial length was reduced to 90 s from baseline session onwards. Even under these conditions, some rats had to be rescued from the water before they found the platform or the 90 s task-time elapsed. We assigned the maximum 90 s value to these animals. During the treatment period the test was repeated twice with the platform located first at NW and then in the center of the maze, the location completely new for the animals. The primary performance parameter was the time to find the target (escape latency); average of the 4 daily trials was used as individual value in the statistical calculation.

#### Cooperation task in the skinner box

The assay is described in detail in^13^. Two rats were placed in the same Skinner box. The opposite walls of the chamber were equipped with one nose-poke module and one magazine for each. In order to obtain food reward, animals had to perform simultaneous nose-pokes after a stimulus light was turned on in both modules. The nose-pokes at the opposite sides were regarded as simultaneous if the delay between them did not exceed 1 s. Non-simultaneous responses or repeated nose-pokes to the same module were punished with 5 s timeout. The animals were trained to learn the task from 13 till 15 months of age according to the scheme described in the SI file. They were then given regular maintenance training sessions until the start of the current study. The outcome parameters were the number of initiated trials, the percentage of successful trials and the number of rewards obtained.

#### Pot jumping test

The test served to measure procedural learning capabilities and was designed according to^14^. Briefly, the experiment was conducted in the MWM tank, where 12 flower pots were placed upside down forming a circle. Distance between the centers of the adjacent pots gradually increased from 18 to 46 cm in an anticlockwise direction. The tank was filled with water to a depth of 6 cm to restrain rats climbing off the pots. During a session, animals were placed onto the start pot and allowed to freely move on the pots for 3 min while their behavior was recorded with a video camera system. The longest inter-pot distance jumped over was the primary performance parameter, but total number of jumps and number of jumps performed until reaching the farthest pot were also registered. The latter value was compared to the theoretically minimum number of jumps needed to reach the same pot and the ratio of the two values served as a jumping efficacy variable. Pot jumping training of the animals started at the age of 7 months and continued with several break periods until the present study.

#### Nose-poke: lever-press discrimination (NP-LP)

This paradigm was first introduced to the animals on the 2nd treatment day and was carried out in the same Skinner-box apparatus where the cooperation paradigm had been carried out, this time also equipped with a lever on one side. The task was the following: when the nose-poke module was lit a nose-poke response resulted in a reward, whereas when the lever lamp was lit a lever-press response was needed to get a pellet. The light signals were on for 10 s in both cases or until the proper response was performed. The difficulty of the task lied in that nose-poking formed part of the rats’ behavioral repertoire but lever pressing was a novel response to be acquired. If the rat did not produce the required response during the 10 s activation period (omission) the lights went off and a 5 s long timeout interval commenced. Following a correct response, the operandum to be activated for the next trial was randomly chosen; however, following an omission response always the same operandum was activated in the next trial as long as the rat did not perform a correct response. The number of trials and correct responses were registered as outcome variables.

#### Motor activity measurement

Spontaneous locomotor activity was measured in an activity monitor (L: 50 cm, W: 50 cm, H: 40 cm) equipped with 2 × 32 pairs of infrared photo beams along all bottom axes of the cage. Animals were placed into the apparatus for 30 min. Motor activity was determined as the total number of beam interruptions during this period.

### Statistical analysis of the behavioral measurements

Behavioral data of each test were analyzed with repeated measures ANOVA with ’treatment’ as the between group factor and ’measurement days’ as repeated measures factor, respectively, and Duncan-test was applied for post-hoc comparisons. All the statistical output tables can be found in the Supplemental Information file.

#### Multivariate analysis of the behavioral results

To get an overall image on the behavioral effect of CE-123 a multivariate analysis of variance was performed on the output variables of the behavioral paradigms measured in the treatment period. Altogether we extracted 17 variables from the six assays and grouped them into 3 types (Table S1): (1) motivational variables were those which reflect the animal’s activity in the particular assay, its inclination to perform the task. Activity in the motimeter and the pot jumping test, number of initiated trials in the operant assays and percentage of missed trials in the five-choice test belong to this group. (2) “Success” variables were: the net results of the sessions, i.e. the number of pellets earned in the operant assays, the number of trials with successful escape in the water-maze, and the longest distance jumped in the pot jumping test (5 variables). (3) Efficacy variables: how efficiently the rat could acquire the rewards. Percentage of rewarded trials out of all in the operant assays, escape latency in the water-maze and the jumping efficacy in the pot jumping test feature this group. We then separately conducted multivariate ANOVAs on these groups of parameters.

### Study design

The flow of the study is summarized in Table 1. First, baseline performance was recorded in the acquired cognitive paradigms parallel with habituating the animals to daily intraperitoneal injections. Based on the results animals were randomly assigned to the treatment groups (*see* SI). CE-123 or vehicle treatment was going on for 15 days, meanwhile testing in the known cognitive paradigms continued and was supplemented with a novel task to learn. The last day, 2.5 h after treatment and following the behavioral measurements animals were sacrificed, blood samples were collected and brains were dissected (*see* SI for details).

**Table 1:** Outline of the study.

Day	Test		Treatment	Remark
-7	Pot jumping test (50-72)		saline ip.	Routine maintenance training baseline performance
-6	MWM-NE (50-60)	5-CSRTT 1s (62-72)	saline ip.	
-5	MWM-NE (62-72)	5-CSRTT 1s (50-60)	saline ip.	
-4	Cooperation task (in pairs)		saline ip.	
-3	Nose-poking with inactive lever (50-72)		saline ip.	Routine training
-2	Lever-press training (50-72)		saline ip.	New learning
-1	Lever-press training (50-72)		saline ip.	
**Randomization to treatment groups**				
1	Motor activity (50-72)		CE-123/vehicle	
2			CE-123/vehicle	
3	Cooperation task (in pairs)		CE-123/vehicle	Standard assay conditions
4	MWM-NW (50-5D)	5-CSRTT 0.25s (5E-72)	CE-123/vehicle	Challenge conditions
5	MWM-NW (5E-72)	5-CSRTT 0.25s (50-5D)	CE-123/vehicle	Challenge conditions
6	NP-LP (50-72)		CE-123/vehicle	Novel task
7			CE-123/vehicle	
8			CE-123/vehicle	
9	NP-LP (50-72)		CE-123/vehicle	Novel task
10	Cooperation task (in pairs)		CE-123/vehicle	Standard assay conditions
11	MWM-center (50-5D)	5-CSRTT 0.25s (5E-72)	CE-123/vehicle	Challenge conditions
12	MWM-center (5E-72)	5-CSRTT 0.25s (50-5D)	CE-123/vehicle	Challenge conditions
13	Cooperation task (in pairs)		CE-123/vehicle	Standard assay conditions
14	Pot jumping test (50-72)		CE-123/vehicle	Standard assay conditions
15	Motor activity (50-72)	NP-LP (50-72)	CE-123/vehicle	Novel task
			sacrifice (blood sampling, brain dissection)	

Numbers in brackets show the hexadecimal identifiers of the animals, for MWM a position of the platform is indicated (NE, NW or center).

### Drug treatment

CE-123 (10 mg/kg) dissolved in 5% DMSO and 7.5% Tween 20 solution or vehicle (2 ml/kg injection volume) were ip administered once a day for 15 days, 60 min before the actual learning task; in case of motor activity measurement the pre-treatment time was 30 min. Separate persons performed the injections and the learning assays; those who did the latter were not aware of which treatment the animals received.

### Quantification of CE-123 in rat plasma and brain

Plasma and brain levels of CE-123 2.5 h after ip administration were measured by liquid chromatography-high resolution mass spectrometer (LC-HRMS). The LC-HRMS system consisted of a Dionex UltiMate 3000 RSLC-series, (Thermo Fisher Scientific, Inc., Germany) coupled to a maXis HD ESI-Qq-TOF mass spectrometer (Bruker Corporation, Germany). The in-house validation was conducted according to the guidelines of the ICH^17^ and the USFDA^18^ by evaluating the following parameters: linearity, accuracy, intra- and inter-day precision, sensitivity and stability. Details regarding LC-HRMS operation parameters as well as the sample preparation protocol and in-house validation are reported in the Supplemental Information.

### Proteomics quantification of synaptosomal PFC fraction

Protein sample preparation and LC–MS/MS was performed as previously described^19^ (for details see SI).

MaxQuant software (version 1.6.17.0)^20^ was used for label-free quantification. Data were searched against *Rattus norvegicus* UniProt sequence database (downloaded on August 18th, 2020; 8112 entries) using the following search parameters: carbamidomethylation of cysteine as fixed modification; oxidation of methionine and protein N-terminal acetylation as variable modifications; trypsin as proteolytic enzyme with maximal two missed cleavages; a second peptide option was used; 20 ppm and 4.5 ppm of peptide mass error tolerances for first search and second search, respectively; minimum of 7 aa per a peptides. For identification parameters were set to: 0.01 PSM FDR; 0.01 protein FDR and 0.01 site decoy fraction. The parameters for the match between runs algorithm was set to: matching time window 0.7 min and alignment time window 20 min.

All data were analysed by Perseus software^21^ as follows. The data set was filtered for proteins with a minimum of 10 valid values (≥ 2 unique peptides) in at least one group (CE-123 or vehicle). LFQ intensities were log2-transformed and missing values were imputed using a downshifted normal distribution (width 0.3, downshift 1.5). Next, a t-test was performed with correction for multiple testing using the Benjamini–Hochberg method. Protein groups with q-value < 0.05 and fold change > 1.5 were considered significantly changed and used for functional analyses.

For the heat map, the log2-transformed LFQ intensities were z-scored and the hierarchical clustering was computed using the euclidean distance. Gene ontology (GO) analysis was performed using the ClueGO (v2.5.7) Cytoscape (3.7.1) plug-in^22^. ClueGO parameters were set as indicated: Go Term Fusion selected; only display pathways with *p*-values ≤ 0.05; GO tree interval 3–10 levels; GO term minimum # genes, 3; threshold of 4% of genes per pathway. The statistical test used for the enrichment was based on right-sided hypergeometric test with a Benjamini–Hochberg correction and kappa score of 0.4.

## Results

### Plasma and brain level of CE-123

The mean ± SEM values of CE-123 concentration in rat plasma and brain tissue 2.5 h after ip administration of 10 mg/kg were 3100 ± 352 ng/mL (n = 8) and 1800 ± 214 ng/g (n = 6), respectively, resulting in a 0.58 brain/plasma level ratio. The results of quantification CE-123 are summarized in the Supplemental Information in Tables S5 and S6.

### Spontaneous motor activity measurement

**Figure 1:**
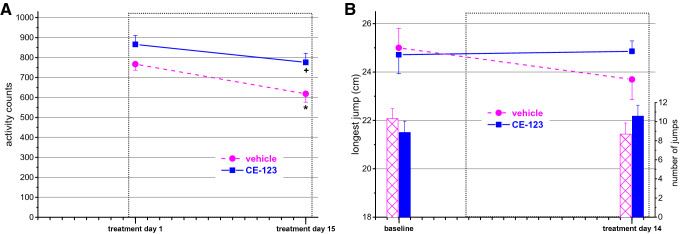
Spontaneous motor activity (**A**) and performance in the pot jumping test (**B**) of animals repeatedly treated with CE-123 or vehicle. (For the study design see “Methods and materials”). Data are expressed as mean ± SEM. Scaling of X-axis reflects calendar days. Dotted rectangle signs the treatment period. (**A**): Activity counts detecting horizontal movements. (**B**): Upper curves, left axis: longest distance jumped; lower columns, right axis: total number of jumps in the trial. *p < 0.05 significance of the difference from the first measurement in the same group. +*p* < 0.05 significance of the difference between groups on the same day (repeated measures ANOVA followed by Duncan test).

ANOVA revealed a significant group effect (F_group_(1,25) = 9.398; *p* < 0.01) and a significant time effect (F_day_(1,25) = 8.135; *p* < 0.01). A single dose of 10 mg/kg CE-123 induced a mild, non-significant increase in motor activity (Fig. 1A). When the test was repeated the activity of the control group significantly decreased reflecting habituation to the test apparatus while that of the drug treated animals non-significantly decreased altogether resulting in a significant but mild (25%) difference between the groups by the end of the treatment period.

### 5-choice serial reaction time test (5-CSRTT)

**Figure 2:**
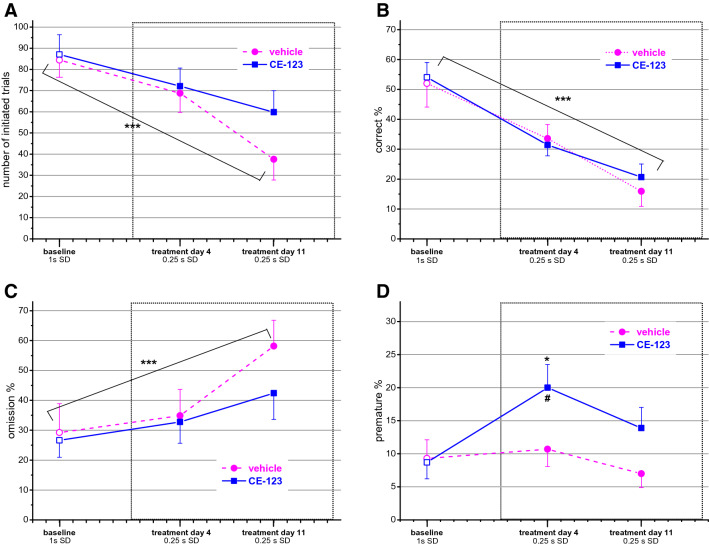
Performance in the five-choice serial reaction time test (5-CSRTT) of animals repeatedly treated with CE-123 or vehicle. (For the study design see “Methods and Materials”). (**A**) Number of initiated trials. (**B**) Percent correct responses. (**C**) Percent omissions (missed responses). (**D**) Percent premature responses. Data are expressed as mean ± SEM. Scaling of X-axis reflects calendar days. Dotted rectangle signs the treatment period. ***p < 0.001 repeated measures effect. *p < 0.05 significance of the difference from the baseline in the same group. #*p* < 0.1 significance of the difference between groups on the same day (repeated measures ANOVA followed by Duncan test); SD: stimulus duration.

In this paradigm, decreasing the stimulus duration (i.e. making the task more difficult) caused a similar reduction in performance in both groups shown by the significant decrease in the number of initiated trials (F_day_(2,50) = 21.15; *p* < 0.001) and the percentage of correct responses (F_day_(2,50) = 41.10; *p* < 0.001) and by a significant increase in the proportion of omitted trials (F_day_(2,50) = 14.25; *p* < 0.001) (Fig. 2A–C). No significant interaction or treatment effect was found on these variables. Interestingly, CE-123 treated animals showed a higher percentage of premature responses (F_group_(1,25) = 3.509, *p* < 0.08; F_day_(2,50) = 3.474; *p* < 0.05) at least at the beginning of the treatment (Fig. 2D).

### Morris water-maze (MWM)

**Figure 3:**
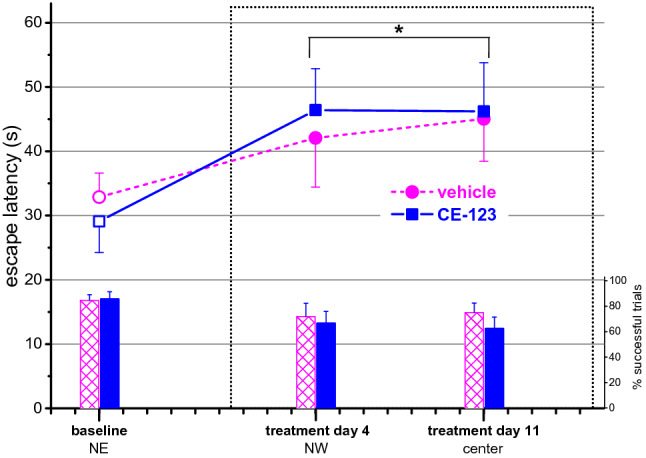
Performance in the Morris water-maze of animals repeatedly treated with CE-123 or vehicle. (For the study design see “Methods and Materials”). Upper curves, left axis: escape latency; lower columns, right axis: percentage of successful escape trials. Data are expressed as mean ± SEM. Scaling of X-axis reflects calendar days. Dotted rectangle signs the treatment period. *p < 0.05 significance of the difference from baseline (repeated measures ANOVA followed by Duncan test); NE, NW, and ‘center’ indicates the position of the escape platform.

In this assay, no difference between the two groups was observed (Fig. 3). ANOVA revealed a significant increase in escape latency (F_day_(2,50) = 4.306; *p* < 0.05) which reflected the increased number of trials when the platform was not found. In the vehicle group 7 animals had to be rescued at altogether 19 occasions (out of 91) during the treatment period while 11 rats at 26 occasions (out of 98) were rescued in the drug-treated group. The average rescue time was 68 s and 65 s in the control and CE-123 groups, respectively.

### Cooperation task in the skinner box

**Figure 4:**
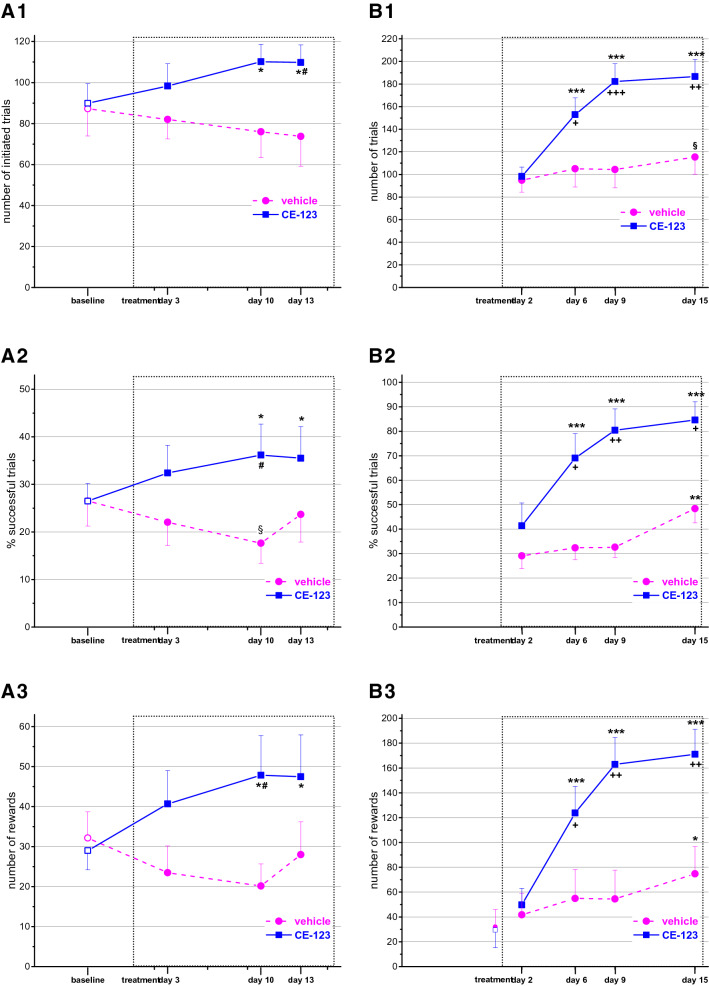
Performance in the cooperation task (**A**) and in the nose-poke—lever-press discrimination paradigm (**B**) of animals repeatedly treated with CE-123 or vehicle. (For the study design see “Methods and Materials”). (**A1**–**A3**): number of initiated trials, percentage of rewarded trials, and number of rewards obtained, respectively. (**B1**–**B3**): number of trials, percentage of rewarded trials, and number of rewards obtained, respectively. The hollow symbols in (**B3**) indicate the number of rewards obtained in the last lever-press session. Data are expressed as mean ± SEM. Scaling of X-axis reflects calendar days. Dotted rectangle signs the treatment period. §, *, **, ***p < 0.1, *p* < 0.05, *p* < 0.01, *p* < 0.001 significance of the difference from the baseline (cooperation paradigm) or treatment day 2 (NP-LP discrimination) in the same group. #, +, ++, +++*p* < 0.1, *p* < 0.05, *p* < 0.01, *p* < 0.001 significance of the difference between groups on the same day.

Statistical evaluation showed significant interaction between the treatment (group effect) and the different measurement points (day effect) affecting the number of initiated trials (F_groupxday_(3,66) = 3.197, *p* < 0.05), the percent successful (rewarded) trials (F_groupxday_(3,66) = 3.459, *p* < 0.05) and the number of rewards obtained (F_groupxday_(3,66) = 3.847, *p* < 0.05). Post-hoc analysis revealed that all the three parameters significantly increased compared to their baseline level in the CE-123 treated group but not in the control animals (Fig. 4A). This deviation resulted in marginally significant difference between the groups in the later trials of the treatment period.

### Nose-poke: lever-press discrimination (NP–LP)

This was a novel task for the animals with 4 acquisition sessions during the treatment period. All three ANOVA terms were significant for the variables, number of trials (F_groupxday_(3,75) = 10.68, *p* < 0.001), percentage of successful trials (F_groupxday_(3,75) = 5.719, *p* < 0.01), and number of rewards obtained (F_groupxday_(3,75) = 10.67, *p* < 0.001). In contrast to the control animals, rats treated with CE-123 rapidly acquired the discrimination task and reached 85% efficacy by the last session. It resulted in obtaining 2–3 times more pellets than the vehicle treated animals (Fig. 4B).

### Pot jumping test

There were not significant differences between the two groups either in the longest distance jumped over or in the total number of jumps (Fig. 1B) or in the jumping efficacy while reaching the farthest pot (Table S1).

### Multivariate analysis of the behavioral results

When the multivariate ANOVA was carried out with all 17 variables the difference between the vehicle and drug treated group was not significant (Table S2). The multivariate ANOVAs separately conducted on the motivational, success and efficacy groups of parameters revealed significant differences between the two groups in each class (Table S2). However, the highly significant differences in the nose-poke—lever-press discrimination paradigm may have distorted the results (when other variables are added to an NP-LP variable the difference in the latter is high enough to pull the others with it), therefore, we repeated the MANOVA in the 3 variable groups excluding the NP-LP parameters. The reward and efficacy variables fell out of the 5% type I error limit, but the motivational variables still showed a significant difference (Table 2).

**Table 2:** Multivariate ANOVA results (Wilks λ) of the structured behavioral variables excluding the NP-LP paradigm (see text for details).

Variables	Wilk’s λ	F (df_effect_, df_error_)	*p* value
Motivational: 5CSRTT-ITI, 5CSRTTomiss%, coop-ITI, PJ-#jumps, hor.act	0.5703	3.1645 (5,21)	0.0277
Success: 5CSRTTrew, cooprew, MWM#esc, PJ-ld	0.7289	2.0452 (4,22)	0.1229
Efficacy: 5CSRTTcorr%, coop-ITI%, MWMlat, PJ-ldeff	0.7438	1.8946 (4,22)	0.1472

5CSRTT-IT, coop-IT: number of initiated trials in the 5-CSRTT and cooperation task, respectively; 5CSRTTomiss%: percent missed trials in the 5-CSRTT; PJ-#jumps: number of jumps in the pot jumping test; hor.act.: activity in the motimeter; 5CSRTTrew, cooprew: number of pellets earned in the 5-CSRTT and cooperation task, respectively; MWM#esc: number of trials with successful escape in the MWM; PJ-ld: longest distance jumped in the pot jumping test; 5CSRTTcorr%, coop-ITI%: percent rewarded trials out of all in the 5-CSRTT and cooperation task, respectively; MWMlat: escape latency in the MWM; PJ-ldeff: jumping efficacy in the pot jumping test.

### Proteomic changes in prefrontal cortex after CE-123 treatment

Label-free quantification was used to identify proteomic differences in prefrontal cortex between two groups of aged rats subchronically administered 10 mg/kg CE-123 (n = 11) and vehicle (n = 13) and trained in several behavioral tasks.

**Figure 5:**
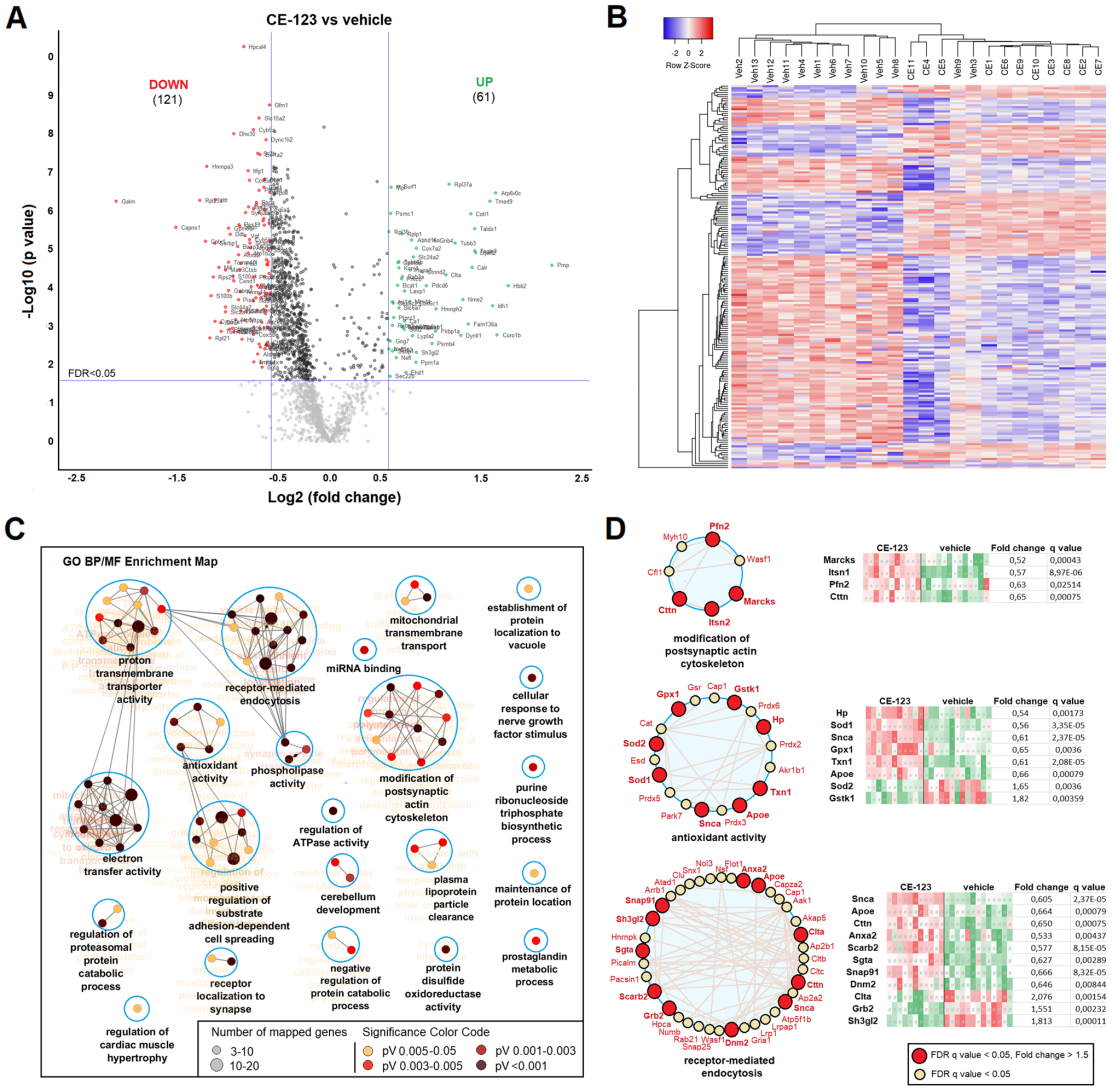
Enriched synaptosomal prefrontal cortex fractions from aged CE-123- and vehicle-treated groups were subjected to label-free quantitative proteomic analysis. (**A**) A volcano plot showing proteins upregulated (green) and downregulated (red) in CE-123 group compared to vehicle (q < 0.05, fold-change > 1.5). The protein groups in the upper middle section (black) are those which fulfilled the requirement for the q-value cut-off but did not match the fold-change cut-off. (**B**) A hierarchical clustering heatmap is presented for the differentially expressed proteins (q < 0.05, fold change > 1.5; n = 11 and 13). (**C**) The clustered network map of enriched gene ontology (GO) Biological Processes (BP) and Molecular Function (MF) terms on the list of significantly altered proteins between the groups. Enriched GO terms consisting of various related genes are depicted as nodes. The size of the nodes represents the number of genes in the set. The FDR q-value of each GO-term is color-coded. Displayed GO terms are the most significant cases from significantly enriched clusters. The connectivity (edges) between the terms in a functionally grouped network is derived from kappa score, which indicates the similarity of associated genes shared by different terms. (**D**) Representative GO terms depicted together with related genes (small circle) significantly altered in the CE-123 group, FDR q-value and fold change are indicated.

A total number of 1693 protein entries were unambiguously identified and used for quantification (> 2 unique peptides in at least 10 biological replicates in at least 1 group). A total number of 182 protein groups were significantly different between treatment groups (FDR q < 0.05, fold-change > 1.5). Of these, 61 proteins were up-regulated and 121 proteins down-regulated in CE-123 group compared to vehicle (Fig. 5A). A hierarchical clustering heatmap shows differently expressed proteins in all samples (Fig. 5B). All significantly different proteins between treatment groups are listed in Supplementary Table.

In an attempt to translate proteomic changes after subchronical treatment with CE-123 into functional consequences, functional enrichment analysis on significantly different proteins was performed using the Cytoscape plug-in ClueGo^22^. 85 significantly enriched GO terms were categorized via their shared genes into 22 GO groups. As shown in Fig. 5C, altered protein groups were enriched in various processes, such as proton transmembrane transport, electron transfer activity, modification of postsynaptic actin cytoskeleton, receptor-mediated endocytosis, antioxidant activity, etc., whereas majority of associated proteins were downregulated in the CE-123 group (Fig. 5D). All significantly enriched GO terms and identified associated proteins are listed in Supplementary Table.

The mass spectrometry data have been deposited to the ProteomeXchange Consortium via the PRIDE^23^ partner repository with the dataset identifier PXD022737.

## Discussion

### Behavioral assays

As dopamine uptake inhibitors are known to have stimulatory effects, measurement of motor activity served on one hand as a behavioral “verification” (biomarker) of the target occupation by the compound, on the other hand to ascertain that the applied dose is not too high to risk the appearance of cognition-disruptive stimulatory effects. The observed mild increase in motor activity provided a reassuring answer to both questions. *Post mortem* measurements gave an approximate of the effective plasma and brain level of the compound providing a 0.58 brain/plasma ratio. In the study of^6^ single dose of 10 mg/kg ip CE-123 yielded 3 μg/mL plasma level and 2 μg/g brain level at 1 h post-dose. Our data obtained at 2.5 h after the 15th dose are very similar to these values, thus we may assume that the compound reached steady state level by the end of the treatment period.

Concerning the eventual unwanted stimulatory effect, the slightly increased activity may indicate a higher exploratory motivation in a novel environment rather than non-specific motor stimulation. Lack of motor activation in the familiar environment of the pot jumping test also makes this assumption likely. Changes in other parameters in the other assays also suggest increased motivation. CE-123 treated animals showed less increase in the percentage of omitted responses in the 5-CSRTT and initiated a higher number of trials indicating that they remained more involved in the task than the controls despite the increased difficulty. An elevated number of initiated trials could be observed in the cooperation and NP-LP assays as well. In fact, in the latter paradigm the key for success (getting more reward) was the ability to “discover” the function of the lever, which implied more explorative activity in the cage (control animals only started to use the lever in the last session). Although none of the above changes reached the 5% statistical significance, with the exception of the NP-LP task, they all pointed to the same direction. That made us to categorize the various parameters of the assays into motivational, success and efficacy classes and carrying out a multivariate analysis on each category. And indeed, the motivational class proved to be significantly different between the two groups even when we left out the NP-LP assay from the analysis to avoid a “one big effect” bias. Our results on motivational function are in accordance with^8^ who described that CE-123 improved performance in two effort-based tasks in rats. Interestingly, modafinil was found to be ineffective in a similar task in rats^24^, although data suggestive for increased motivation by modafinil were published on depressed patients^25^ as well as healthy volunteers^2,26^.

However, CE-123 was not able to change the disrupted attentional performance caused by the decrease in stimulus duration in the 5-CSRTT. This finding is in accordance with that of^7^ who obtained similar results (i.e. lack of effect) for CE-123 and modafinil, too. Clinical data, albeit somewhat controversial, also do not indicate a marked effect of modafinil on attention^2,3,27^.

It is of particular note that a marginally significant increase in premature responding (indicating increased impulsivity) was observed in the 5-CSRTT. The effect, however, was transient as it substantially diminished by the end of the treatment period. This finding is in contrast to that of^7^ where the compound did not affect premature responding. However, they used young animals and from a different strain, applied acute dosing of the compound and the baseline level of premature responses was relatively high due to the long inter-trial interval applied. Our results better resemble the findings obtained with modafinil, which also increased premature responses in rats at shorter stimulus durations^28,29^. Moreover, modafinil also increased impulsivity in touchscreen continuous performance task^30,31^, gambling task^32^ and go-signal task^33^ when the baseline was low, but decreased^33,34^ or did not affect it^31^ when the baseline was high. In humans a baseline dependent action of modafinil on impulsivity was also observed in pathological gamblers^35^: increase in patients with low impulsivity and decrease in patients with high impulsivity. The symptom improvement by modafinil in ADHD children^5,36^, who have high baseline impulsivity, also fits this profile, however, modafinil decreased impulsivity in healthy volunteers^1^, sleep-deprived doctors^4^, and first episode schizophrenics^27^ as well. As far as CE-123 is concerned, further, more specific experiments are needed to fully characterize its effect on impulsivity but based on the results obtained so far, we predict actions in humans similar to those of modafinil.

In the cooperation assay the compound increased not only the initiated trials but also the proportion of the successful trials. This task requires a synchronized behavior from the members of the pair, so besides the increased motivation, better cooperation also contributed to the higher success. It suggests that CE-123 may be effective on social cognitive dysfunctions. This presumption is further confirmed by the finding of^9^ in mice where the compound showed a protective effect against retroactive interference in social recognition. The pharmacological congener modafinil also showed effects on social cognition: it restored the chronic restrain stress-induced decrease in social discrimination though it had no effect in non-restrained rats^37^. Nevertheless, in humans modafinil improved recognition of sad faces in first episode schizophrenics^38^, and significantly improved the social cognition domain of the Matrix consensus cognitive battery in healthy volunteers^39^.

CE-123 had no effect on navigational performance in the MWM. In contrast to this finding, in a hole-board paradigm of spatial learning^6^ a single dose of the compound produced improvement in performance. Findings with modafinil in this paradigm, though not entirely consistent, demonstrate an improving action, too^40–43^. However, in our study the MWM was a very well learnt task, in which the animals, those which did not have swimming difficulties, proficiently navigated, that may have given rise to a ceiling effect for an eventual improvement. Other results from the MWM assay suggest that CE-123 did not have a positive effect on the physical condition of the animals: if the compound had enhanced the physical condition, we could have seen fewer animals rescued, at fewer occasions and with longer rescue time compared to the controls; but this was not the case. Interestingly, there are human data with modafinil showing it reduced the subjective feeling of fatigue and increased that of vigor in narcoleptic patients^44^.

Summarizing the behavioral pattern of CE-123 observed in the test system with aged rats, it can be characterized by markedly enhanced motivation which resulted in superior performance in a new-to-learn operant task and in a cooperation assay, and by a signal of mildly increased impulsivity. The compound did not affect attention, spatial and motor learning. Based on our results, we can speculate that CE-123, a low affinity DAT inhibitor superior to the parent compound modafinil in terms of affinity and specificity, will at least partially resemble effects observed in humans for modafinil. A direct comparison of the two compounds in the test system would verify this assumption. However, this was not possible as we had a limited number of aged experienced animals.

### Proteomics

Several protein groups associated with aging and neurodegenerative diseases including apolipoprotein E (ApoE), S100B, Sod1, Sod2, huntingtin (Htt), macrophage migration inhibitory factor (Mif), α-Synuclein (Snca) were modulated by either drug treatment or the behavioural training per se. For instance, we detected more than a twofold decrease of S100B protein levels in a synaptosomal fraction of PFC. There is increasing evidence that S100B acts as a pro-inflammatory cytokine or damage-associated molecular pattern factor depending on its concentration. Lower concentrations of extracellular S100B act on glial and neuronal cells as a growth differentiating factor, while higher concentrations induce apoptosis^45^. Increased S100B and MAP-2 levels promote dendritic maturation and leads to the loss of dendrites^46^. Elevated levels of S100B have been detected in various clinical conditions that include inflammatory, neurodegenerative and psychiatric disorders^47–49^. Protein groups annotated to GO terms “dendritic spine organisation”, “dendritic spine morphology”, etc. grouped to “modification of postsynaptic actine cytoskeleton” were also altered by CE-123 treatment (Fig. 5C, D). Decrease of synaptic levels of S100B after CE-123 treatment may be one of the contributing factors to superior behavioural performance.

α-Synuclein, also down-regulated in the CE-123 group, is a presynaptic protein that under physiological conditions associates with synaptic vesicle membranes and regulates synaptic vesicle trafficking^50^. However, increased levels of α-synuclein lead to formation of aggregates and produce a physiological defect in synaptic vesicle recycling, which is associated with compromised neurotransmission^51,52^ and linked to Parkinson’s disease and other neurodegenerative diseases^50,53^. The GO terms cluster “receptor-mediated endocytosis” comprising of GO terms like synaptic vesicle recycling, synaptic vesicle transport, receptor internalization, etc., was one of the most prominent processes modulated by CE-123 treatment (Fig. 5D). There is evidence, that in dopaminergic neurons the presence of DAT influences cellular localization of α-synuclein and DAT-mediated increase in the membrane localization of α-synuclein and in turn may alter DAT activity^54^. Even moderately increased levels of α-synuclein may lead to dysregulation of dopaminergic neurotransmission and increased vulnerability of dopaminergic neurons. We speculate that binding of a specific DAT inhibitor to DAT may modulate DAT-activity altered through interaction with α-synuclein.

Oxidative stress is a key mechanism of the aging process that can cause direct damage to cellular architecture within the brain. Antioxidant enzymes within the brain, such as superoxide dismutase, glutathione peroxidase, glutathione-S-transferase and catalase are critical for breaking down the harmful end products of oxidative phosphorylation^55^. Antioxidant activity belong to the processes significantly enriched in functional analysis and modulated by CE-123 treatment (Fig. 5D).

It remains to be shown that the individual significant level changes of proteins from a synaptosomal comparative proteome are causally involved in either altered cognitive processes or can be considered as a result of the pharmacological intervention. This question could have been better answered if the study had involved a group that received only CE-123 treatment without any behavioral test intervention. Unfortunately, due to the low number of available subjects it was not feasible, which is a limitation of the study. The results themselves, however, may help to understand previous work on these proteins and for the design of future studies on the subject of cognitive enhancement and memory function.

A further possible continuation of the study can be its replication in female rats. As CE-123 has only been tested in male animals so far, we have no data on its effects in the other sex. However, based on our knowledge on the actions of modafinil^56,57^ we cannot exclude sex specific effects by CE-123, either.

In summary, the cognitive activity pattern of CE-123 (increased motivation, impulsivity, social cognition) revealed by our complex test system seems to hold relevant information on the expected cognitive profile in the clinic. Mechanistically, the procognitive effects of the compound maybe related to processes of synaptic plasticity and antioxidant activity as proposed by the proteomic outcome.

## Supplementary Information

Supplementary Information 1.

Supplementary Information 2.
