# Supplementary material for: Cognitive profiling and proteomic analysis of the modafinil analogue S-CE-123 in experienced aged rats
Source: Sci Rep. 2021 Dec 14;11:23962. doi: 10.1038/s41598-021-03372-y (PMC8671572; doi:10.1038/s41598-021-03372-y)
Supplement: Supplementary file 1 — Supplementary Information 1. [file 41598_2021_3372_MOESM1_ESM.pdf]

Supplementary information

Cognitive profiling and proteomic analysis of the modafinil analogue S-CE-123 in experienced aged rats

István Gyertyán,[1]* Jana Lubec,[2] Alíz Judit Ernyey,[1] Christopher Gerner,[3] Ferenc Kassai,[1] Predrag Kalaba,[4] Kata Kozma,[1] Iva Cobankovic,[4] Gábor Brenner,[1] Judith Wackerlig,[4] Eva Franschitz[4], Ernst Urban,[4] Thierry Langer,[4] Jovana Malikovic,[5] Gert Lubec[2]*

1: Department of Pharmacology and Pharmacotherapy, Semmelweis University, Budapest, Hungary

2: Programme of Proteomics, Paracelsus Private Medical University, Salzburg, Austria.

3: Department of Analytical Chemistry, University of Vienna, Vienna, Austria

4: Department of Pharmaceutical Chemistry, University of Vienna, Vienna, Austria

5: Core Unit of Biomedical Research, Division of Laboratory Animal Science and Genetics, Medical University of Vienna, Himberg, Austria

Section 1: Behavioral measurements

Materials and methods

Animals

Twenty-seven male Lister hooded rats (Charles River, Germany) of 26 months age at the start of the study were used. They had a prior history of acquiring and routinely performing several learning tasks. The learning history of the animals is detailed at the description of each cognitive paradigm. The average body mass of the animals was 431 g (range: 385-483 g) at the beginning and 442 g (range: 386-500 g) at the end of the study. Animals were housed in groups of three in plastic cages (43x32x21 cm) with wire grid top in a light controlled room (reversed 12-h light/dark cycle, light on at 16:00 h). For environmental enrichment aspen bricks and cardboard tubes were placed in the cages. Rats were fed with commercial pellet rat feed R/M-Z+H produced by SSniff Spezialdiäten GmbH. Daily food intake was limited to 40-45 g per cage. Animals were fed at the end of the active (dark) phase at 15:30, 1-3 hours following their daily training. Access to tap water was ad libitum. The animals were identified by hexadecimal numbers (from 50 to 72) marked on their tail. Rats were intensively handled before and during their behavioral testing.

Learning paradigms

5-choice serial reaction time test (5-CSRTT)

The operant chamber (TSE, Germany) was equipped with five nose-poke modules. Animals were trained to nose-poke into a randomly chosen hole marked for 1 s. In half of the animals, the ‘classical’ 5CSRTT paradigm was applied, where turning on the stimulus light served as a signal (“light on” version). For the rest of the population, a novel, ‘reversed’ 5CSRTT method was used. Here all the nose-poke modules were illuminated, and turning off the stimulus light in one of the holes was the signal (“light off” version). This arrangement originally aimed at testing the assumption that the detection of the ‘off’ signal is more difficult, therefore the attentional load is higher compared to the ‘classical’ 5CSRTT version.

In both paradigms, correct responses were rewarded with a pellet (45 mg purified dustless precision pellets, Bio-Serv) delivered into the magazine. The animal made a premature

response if nose-poked into any of the holes during the 5 s long inter-trial interval, an incorrect response if nose-poked into one of the non-signaled holes, and an omission if it did not respond to the stimulus during its duration plus a 5 s long hold period. Incorrect and premature responses as well as omissions were punished with a 5 s time-out period when the house light was turned off (“light on” version) or on (“light off” version). Nose-poke into the magazine initiated the next trial in each case. Duration of a daily test session was 20 min. Rats acquired the 5-CSRTT through training stages with gradually increasing difficulty until reaching the final test conditions. Half of the animals learned the task in the period of their 6-8 months age (“light on” group), the other half at 8-10 months age (“light off” group). When the latter group also completed the acquisition training, both groups took part in sessions with increased task difficulty when the stimulus duration (SD) was decreased to 0.5 s and 0.25 s. This modification increases the attentional load of the task, resulting in decreased correct responses (Robbins, 2002). Our results showed that the “off” version was a little bit more difficult than the „on” version: it took 5 days more to the animals to acquire the task (defined as at least 40 pellets earned during a session), and the decrease in stimulus duration caused a slightly greater impairment in performance. However, afterwards the two groups showed similar performance. Following this initial learning period rats participated in regular maintenance training involving one session a week until the start of this study. Here, again, task difficulty was increased by reducing the stimulus duration to 0.25 s. The outcome parameters were the following: number of initiated trials, number of rewards obtained (equals number of correct responses), % correct response ratio (correct responses/(total trials-premature responses) x 100), % omission ratio (omissions/(total trials-premature responses) x 100). % premature response ratio (premature responses/total trials x 100). Results of the “on” and the “off” group were pooled as no difference was observed in their performance.

Morris water-maze (MWM)

The task of the animals was to find a hidden 10 cm diameter platform in a 190 cm diameter, 60 cm deep circular tank filled with 39 cm water (23 ± 1 °C). The platform was 1 cm under the water surface, in the south east quadrant, at about 40 cm distance from the side wall of the pool. On the wall of the experimental room extra-maze cues were placed in order to facilitate the orientation during swimming. Animals were trained on four consecutive days in three daily training trials with 30 minutes inter-trial intervals. They were placed in the water at the north, east, south or west edge of the pool in systemic rotation and were given 180 s to escape to the hidden target. They were allowed to remain on the platform for 30 s, afterwards were taken out, dried by a cloth and returned to their cage. Movement of animals was recorded with Smart v3.0 video tracking system software (Panlab, Spain).

Animals were got acquainted with the MWM paradigm at the age of 3 months (n=10) or 6.5-7 months (n=17). At the age of 3-5 and 9-11 months (first 10 animals) or 9.5-11 months (second 17 animals) they all went through a modified version of the task designed to measure a kind of episodic memory (1). From the age of 20 months they underwent maintenance training sessions (4 trials in each) with biweekly frequency and always changing platform location (rotating among quarters south-east (SE), north-east (NE), north-west (NW), south-west (SW)). This routine lasted until the current study. The last such session (platform located at NE) was carried out 5-6 days before the start of the drug treatment period and served as the baseline measurement. However, as the animals were getting older swimming for longer intervals exhausted them so the trial length was reduced to 90 seconds from this baseline session onwards. Even under these conditions rats had to be rescued from the water time to time before they found the platform or the 90 sec task-time elapsed. We assigned the maximum 90 sec value to these animals. During the treatment period the test was repeated

twice with the platform located first at NW and then in the center of the maze – the latter location was completely new for the animals. The primary performance parameter was the time to find the target (escape latency); daily average of the 4 trials was used as individual value in the statistical calculation.

Cooperation task in the Skinner box

The assay is described in details in (2). Two rats were placed in the same Skinner box (MedAssociates, USA). The opposite walls of the chamber were equipped with one nose-poke module and one magazine for each. In order to obtain food reward, animals had to perform simultaneous nose-pokes after a stimulus light was turned on in both modules. The nose-pokes at the opposite sides were regarded as simultaneous if the delay between them did not exceed 1 s. Non-simultaneous responses or repeated nose-pokes to the same module were punished with 5 s timeout. Rats were trained for the task in stages with gradually decreasing intervals allowed for the "simultaneous" nose-pokes from 10 s to 1 s. The training was done in three pairings. First, two cage-mates were trained, which were unfamiliar with the task (naïve-naïve, A+B). After that, the third animal naïve to the task formed a pair with one of its experienced cage-mates (naïve-experienced, C+B). Finally the last remaining combination of the cage-mates worked together (experienced-experienced, A+C). The animals were trained to learn the task from 13 till 15 months of age according to the scheme described above. Three animals did not accomplish the training, so altogether we had 24 cooperating rats. They were given regular maintenance training sessions with biweekly frequency on average until the start of the current study.

At the beginning of the current study a refreshing / baseline training session was held 4 days before the first drug day. Each animal practiced with its familiar partner, and the score of the pair was assigned to each individual. This value was used in calculating group means and in the randomization procedure. Because of the randomized individual treatment allocation, four pairs had to be separated and reunited in another composition for testing in the treatment period.

The outcome parameters were the number of initiated trials, the percentage of successful trials and the number of rewards obtained.

Pot jumping test

The test served to measure procedural learning capabilities and was designed according to (3). Briefly, the experiment was conducted in the MWM tank, where 12 flower pots (16 cm high and 10 cm wide at the bottom) were placed upside down forming a circle. Distance between the centers of the adjacent pots gradually increased from 18 to 46 cm in anticlockwise direction. The tank was filled with 6 cm deep water to restrain rats climbing off the pots. During a session, animals were placed onto the start pot, which was within the shortest distance from the next pot. For 3 minutes they could freely move on the pots and their behavior was observed and recorded with a video camera system. The longest inter-pot distance jumped over was the primary performance parameter, but total number of jumps and number of jumps performed until reaching the farthest pot were also registered. A jumping efficacy variable was also calculated defined as the minimum number of jumps needed to reach the farthest pot reached by the rat in the actual trial divided by the number of jumps actually done before the animal performed its longest jump.
Pot jumping training of the animals started at 7 months age with 7 sessions during two weeks then continued after a 3.5 months break at the age of 11 months. Rats took part in once a week

sessions consisting of two trials a day until their age of 13 months. This was the most intense period of training and the animals reached their „personal best" results during this period. In the next two months the weekly frequency remained but with only one trial a day. Then after a longer break at the age of 19 months they resumed the pot-jumping training with once a month sessions until the present study. In this task, no particular challenge was applied to increase task difficulty as the paradigm inherently offered to choose more difficult (i.e. longer) jumps.

Lever press – nose poke discrimination (LP-NP)

This test was carried out in the same Skinner-box apparatus where the cooperation paradigm had been carried out this time also equipped with a lever on one side. Thus, rats were familiar with the apparatus and the nosepoke hole but they had never met the lever as an operandum. Nose-poking formed part of their behavioral repertoire as both in the cooperation paradigm and in the 5-CSRT task it was the required operant response to get reward, however lever pressing was a novel response to be acquired. Rats got acquainted with the lever in three consecutive daily sessions according to the following: three days before the start of the drug treatment period the animals were placed into the operant chambers where they could obtain a food pellet if nosepoked into the lit nosepoke module. In this session the lever was already present but was inactive. On the next two days the lever was activated, signaled by a lamp lit above the lever, and a leverpress resulted in delivering a food pellet. The nose-poke module was dark and inactive. The nosepoke – lever press discrimination paradigm was first introduced to the animals on the 2nd treatment day. The task was simple: when the nosepoke module was lit a nosepoke response resulted in a reward, whereas when the lever lamp was lit a leverpress response was needed to get a pellet. The light signals were on for 10 seconds in both cases or until the proper response was performed. Inadequate responding, *i.e.* leverpressing under nosepoke module activation or nosepoking under lever activation had no programmed consequences. If the rat did not produce the required response during the 10 s activation period (omission) the lights went off and a 5 s long timeout interval commenced. Following a correct response the operandum to be activated for the next trial was randomly chosen; however, following an omission response always the same operandum was activated in the next trial as long as the rat did not perform a correct response. The intertrial interval was 2 seconds during which neither nosepoking nor leverpressing had programmed consequences. Number of trials and correct responses were registered as outcome variables.

Motor activity measurement

Spontaneous locomotor activity was measured in a three channel activity monitor working with infrared photobeams. Animals were individually placed in the experimental cages (43cm x 43cm x 32cm), and horizontal movements were recorded for 30 min. Motor activity was determined as the total number of beam interruptions during this period. Means ± S.E.M. of activity counts were calculated in each treatment group.

Statistical analysis of the behavioral measurements

Behavioral data were analyzed with repeated measures ANOVA with 'treatment' as the between group factor and 'measurement days' as repeated measures factor, respectively, and Duncan-test was applied for post-hoc comparisons. The Statistica software (version 13.5.0.17)

was used. The statistical output tables can be found in this Supplemental Information document in the 'Statistical tables for the behavioral results' section.

Multivariate analysis of the behavioral results

To get an overall image on the effect of S-CE-123 a multivariate analysis (MANOVA) was performed on the output variables of the behavioral paradigms measured in the treatment period (Table S1). We extracted altogether 17 variables from the six assays and grouped them into 3 types: 1. motivational variables are those which reflect the animal's activity in the particular assay, how much it is involved in it, its inclination to perform the task. Activity in the motimeter (hor.act.) and the pot jumping test (PJ-#jumps), number of initiated trials in the operant assays (NPLP-IT, coop-IT, 5CSRTT-IT) and percent missed trials in the five-choice test (5CSRTTomiss%) belong to this group (6 variables). 2. „Success" variables: the net results of the sessions, i.e. the number of pellets earned in the operant assays (NPLPrew, cooprew, 5CSRTTrew), the number of trials with successful escape in the water-maze (MWM#esc), and the longest distance jumped in the pot jumping test (PJ-ld) belong here (5 variables). 3. Efficacy variables: how efficiently the rat could acquire the rewards. Percent rewarded trials out of all in the operant assays (NPLP-IT%, 5CSRTTcorr%, coop-IT%), escape latency in the water-maze (MWMlat, how quickly they could get out of the water) and the jumping efficacy (PJ-ld eff) feature this group (5 variables). We then separately conducted multivariate ANOVAs on these groups of parameters (Table S2).

Randomization to treatment groups

Animals were randomly assigned to vehicle- and drug treated groups based on their previous performance. Learning parameters (see below) under baseline conditions from all the assays were taken into account by an in-house made algorithm.

- 5CSRTT - % correct response in the baseline session
- 5CSRTT - % omission response in the baseline session
- pot jumping – longest distance jumped in the pre-treatment (baseline) trial
- cooperation task – % successful trials in the pre-treatment session
- MWM – mean escape latency in the session preceding the baseline session (platform SW)
- MWM – mean escape latency in the baseline session (platform NE)
- LP–NP - total number of lever presses in the second lever press session
- body mass of the animals

Drug treatment

S-CE-123 was synthesized in the laboratory of G.L. at the Dept. of Pharmaceutical Chemistry, University of Vienna. The compound was dissolved in 5% DMSO and 7.5% Tween 20 solution (5mg/ml concentration, water clear solution) freshly prepared and used up within two hours. A week before the actual drug treatment a daily ip. treatment regime started to habituate the rats to the injection procedure. Saline was given after the daily maintenance / baseline learning sessions and before feeding the animals. During the drug treatment period 10 mg/kg S-CE-123 or vehicle (2 ml/kg injection volume) were ip.administered once a day for 15 days, 60 minutes before the actual learning task; in case of motor activity measurement

the pre-treatment time was 30 min. Separate persons performed the injections and the learning assays; those who did the latter were not aware of which treatment the animals received.

Blood sampling, brain dissection, sacrificing the animals

On the last treatment day, 2.5 hours after the treatment the animals were sacrificed. Anesthesia was induced by 5% isoflurane and maintained by 2% isoflurane. Whole blood was collected in ACD tubes from caudal vena cava when pedal withdrawal reflex was absent. Plasma was isolated by centrifugation at 2500 rcf at 4°C for 15 min. The supernatant was transferred into 15 mL tubes. The plasma was re-centrifuged to remove remaining debris and to minimize contamination and then put on dry ice for transportation. Upon arrival samples were stored in aliquots at −80°C.

Extraction of brain tissue: immediately following blood sampling decapitation was done by using a sharpened guillotine. Using rongeurs, the skull plate was carefully removed and the brain was taken out using a spatula. The brains were snap frozen in liquid nitrogen then rolled into tinfoil, put into 50 mL falcon tube and then on dry ice for transportation. Upon arrival, the brains were placed on a para cooler (RWW Medizintechnik, Germany) at 6◦C and the prefrontal cortex was removed and finally transferred into precooled cryogenic tubes and stored at -80◦C until tissue was being processed.

Statistical tables for the behavioral results

Spontaneous motor activity

ANOVA table

Effect	Repeated Measures Analysis of Variance (horizontal_all_animals in ce123_moti) Sigma-restricted parameterization Effective hypothesis decomposition; Std. Error of Estimate: 153,2564				
	SS	Degr. of Freedom	MS	F	p
Intercept	30877172	1	30877172	1314,621	0,000000
Group	220729	1	220729	9,398	0,005155
Error	587188	25	23488		
DAY	191750	1	191750	8,135	0,008589
DAY*Group	11532	1	11532	0,489	0,490740
Error	589309	25	23572		

Duncan-test

Cell No.	Duncan test; variable DV_1 (horizontal_all_animals in ce123_moti) Approximate Probabilities for Post Hoc Tests Error: Between; Within; Pooled MSE = 23530,, df = 50,000					
	Group	DAY	{1} 766,97	{2} 618,46	{3} 865,68	{4} 775,66
1	cont	Total		0,018993	0,120522	0,883679
2	cont	Total2	0,018993		0,000260	0,014021
3	ce123	Total	0,120522	0,000260		0,140655
4	ce123	Total2	0,883679	0,014021	0,140655	

Pot-jumping: Longest distance jumped

ANOVA table

Effect	Repeated Measures Analysis of Variance (PJTstat in PJT_ce123_stat_javGyl) Sigma-restricted parameterization Effective hypothesis decomposition; Std. Error of Estimate: 3,1119				
	SS	Degr. of Freedom	MS	F	p
Intercept	32543,49	1	32543,49	3360,558	0,000000
Treatment	2,60	1	2,60	0,269	0,608577
Error	242,10	25	9,68		
R1	4,57	1	4,57	1,010	0,324631
R1*Treatment	7,09	1	7,09	1,566	0,222432
Error	113,24	25	4,53		

Pot-jumping: Total number of jumps

ANOVA table

Effect	Repeated Measures Analysis of Variance (PJTstat in PJT_ce123_stat_javGyl) Sigma-restricted parameterization Effective hypothesis decomposition; Std. Error of Estimate: 5,2715				
	SS	Degr. of Freedom	MS	F	p
Intercept	4977,212	1	4977,212	179,1100	0,000000
Treatment	0,619	1	0,619	0,0223	0,882550
Error	694,714	25	27,789		
R1	0,033	1	0,033	0,0045	0,947168
R1*Treatment	37,366	1	37,366	5,0779	0,033252
Error	183,967	25	7,359		

Duncan-test

Cell No.	Duncan test; variable DV_1 (PJTstat in PJT_ce123_stat_javGyl) Approximate Probabilities for Post Hoc Tests Error: Between; Within; Pooled MSE = 17,574, df = 37,373					
	Treatment	R1	{1} 10,308	{2} 8,6923	{3} 8,8571	{4} 10,571
1	cont	no. jumps_bl		0,155836	0,374875	0,871232
2	cont	no. jumps_tr	0,155836		0,919341	0,297314
3	ce123	no. jumps_bl	0,374875	0,919341		0,132720
4	ce123	no. jumps_tr	0,871232	0,297314	0,132720	

Pot-jumping: Efficacy in reaching the farthest pot

ANOVA table

Effect	Repeated Measures Analysis of Variance (PJTstat in PJT_ce123_stat_javGyl) Sigma-restricted parameterization Effective hypothesis decomposition; Std. Error of Estimate: 27,2022				
	SS	Degr. of Freedom	MS	F	p
Intercept	208899,9	1	208899,9	282,3127	0,000000
Treatment	1611,8	1	1611,8	2,1782	0,152465
Error	18499,0	25	740,0		
R1	56,1	1	56,1	0,1512	0,700681
R1*Treatment	364,8	1	364,8	0,9826	0,331047
Error	9282,4	25	371,3		

5-CSRTT (5-choice serial reaction time test):_Number of initiated trials

ANOVA table

Effect	Repeated Measures Analysis of Variance (5CSRTT in ce123_5CSRTT) Sigma-restricted parameterization Effective hypothesis decomposition; Std. Error of Estimate: 50,5258				
	SS	Degr. of Freedom	MS	F	p
Intercept	377281,1	1	377281,1	147,7878	0,000000
Treatment	1790,7	1	1790,7	0,7015	0,410228
Error	63821,4	25	2552,9		
DAY	18701,9	2	9351,0	21,1465	0,000000
DAY*Treatment	1647,3	2	823,6	1,8626	0,165882
Error	22110,0	50	442,2		

5-CSRTT: % correct responses

ANOVA table

Effect	Repeated Measures Analysis of Variance (5CSRTT in ce123_5CSRTT) Sigma-restricted parameterization Effective hypothesis decomposition; Std. Error of Estimate: 26,4925				
	SS	Degr. of Freedom	MS	F	p
Intercept	96925,96	1	96925,96	138,1001	0,000000
Treatment	48,84	1	48,84	0,0696	0,794100
Error	17546,33	25	701,85		
DAY	16436,52	2	8218,26	41,1000	0,000000
DAY*Treatment	160,76	2	80,38	0,4020	0,671141
Error	9997,88	50	199,96		

5-CSRTT: % omissions

ANOVA table

Effect	Repeated Measures Analysis of Variance (5CSRTT in ce123_5CSRTT) Sigma-restricted parameterization Effective hypothesis decomposition; Std. Error of Estimate: 46,8264				
	SS	Degr. of Freedom	MS	F	p
Intercept	112842,7	1	112842,7	51,46255	0,000000
Treatment	934,4	1	934,4	0,42612	0,519856
Error	54817,9	25	2192,7		
R1	7230,2	2	3615,1	14,24855	0,000013
R1*Treatment	808,8	2	404,4	1,59397	0,213266
Error	12685,8	50	253,7		

5-CSRTT: % premature responses

ANOVA table

Effect	Repeated Measures Analysis of Variance (5CSRTT in ce123_5CSRTT) Sigma-restricted parameterization Effective hypothesis decomposition; Std. Error of Estimate: 12,4215				
	SS	Degr. of Freedom	MS	F	p
Intercept	10875,43	1	10875,43	70,48494	0,000000
Treatment	541,38	1	541,38	3,50875	0,072784
Error	3857,36	25	154,29		
R1	591,35	2	295,67	3,47419	0,038656
R1*Treatment	359,20	2	179,60	2,11033	0,131865
Error	4255,29	50	85,11		

Duncan-test

Cell No.	Duncan test; variable DV_1 (5CSRTT in ce123_5CSRTT) Approximate Probabilities for Post Hoc Tests Error: Within MSE = 85,106, df = 50,000			
	R1	{1}<br>8,9837	{2}<br>15,495	{3}<br>10,594
1	prem0		0,016558	0,524337
2	prem1	0,016558		0,056660
3	prem2	0,524337	0,056660	

Duncan-test

Cell No.	Treatment	R1	{1} 9,3051	{2} 10,682	{3} 7,0371	{4} 8,6853	{5} 19,965	{6} 13,897
			Duncan test; variable DV_1 (5CSRTT in ce123_5CSRTT) Approximate Probabilities for Post Hoc Tests Error: Between; Within; Pooled MSE = 108,17, df = 68,749					
1	cont	prem0		0,700115	0,552641	0,877584	0,015835	0,285363
2	cont	prem1	0,700115		0,357899	0,642702	0,030542	0,425162
3	cont	prem2	0,552641	0,357899		0,682161	0,004583	0,132371
4	ce123	prem0	0,877584	0,642702	0,682161		0,005243	0,188170
5	ce123	prem1	0,015835	0,030542	0,004583	0,005243		0,094044
6	ce123	prem2	0,285363	0,425162	0,132371	0,188170	0,094044	

5-CSRTT: % accuracy

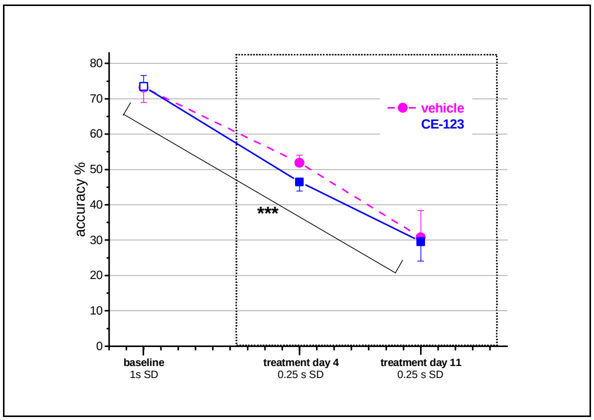

Figure S1. Accuracy performance (=correct responses/(correct + incorrect responses) in the five-choice serial reaction time test (5-CSRTT) of animals repeatedly treated with S-CE-123 or vehicle. (For the study design *see* Methods and Materials.) Shown are mean ±SEM values. Scaling of X-axis reflects calendar days. Dotted rectangle signs the treatment period. ***: p<0.001 significant ‘day’ effect (repeated measures ANOVA); SD: stimulus duration

ANOVA table

Effect	SS	Degr. of Freedom	MS	F	p
	Repeated Measures Analysis of Variance (5CSRTT in ce123_5CSRTT) Sigma-restricted parameterization Effective hypothesis decomposition; Std. Error of Estimate: 15,9919				
Intercept	186607,7	1	186607,7	729,6752	0,000000
Treatment	53,1	1	53,1	0,2075	0,653161
Error	5626,3	22	255,7		
DAY	22852,6	2	11426,3	48,1545	0,000000
DAY*Treatment	143,0	2	71,5	0,3014	0,741297
Error	10440,5	44	237,3		

Morris water-maze: Escape latency

ANOVA table

Effect	Repeated Measures Analysis of Variance (statba_MWM in ce123_MWM) Sigma-restricted parameterization Effective hypothesis decomposition; Std. Error of Estimate: 28,0862				
	SS	Degr. of Freedom	MS	F	p
Intercept	131205,8	1	131205,8	166,3285	0,000000
Treatment	6,6	1	6,6	0,0084	0,927811
Error	19720,9	25	788,8		
R1	3523,3	2	1761,7	4,3060	0,018819
R1*Treatment	226,7	2	113,3	0,2770	0,759187
Error	20456,1	50	409,1		

Duncan-test

Cell No.	Duncan test; variable DV_1 (statba_MWM in ce123_MWM) Approximate Probabilities for Post Hoc Tests Error: Within MSE = 409,12, df = 50,000			
	R1	{1} 30,905	{2} 44,312	{3} 45,639
1	Lat (NE) D1 mean		0,018600	0,013487
2	Lat (NW) D2 mean2	0,018600		0,810692
3	Lat (center) D3 mean3	0,013487	0,810692	

Morris water-maze: % successful escape trials

contingency table

	2 x 2 Table (rescue_time_MWM in ce123_MWM)		
	Column 1	Column 2	Row Totals
Frequencies, row 1	10	42	52
Percent of total	9,259%	38,889%	48,148%
Frequencies, row 2	15	41	56
Percent of total	13,889%	37,963%	51,852%
Column totals	25	83	108
Percent of total	23,148%	76,852%	
Chi-square (df=1)	,87	p= ,3523	
V-square (df=1)	,86	p= ,3546	
Yates corrected Chi-square	,49	p= ,4828	
Phi-square	,00801		
Fisher exact p, one-tailed		p= ,2419	
two-tailed		p= ,3723	

Morris water-maze: Escape latency without rescued trials and rescue time

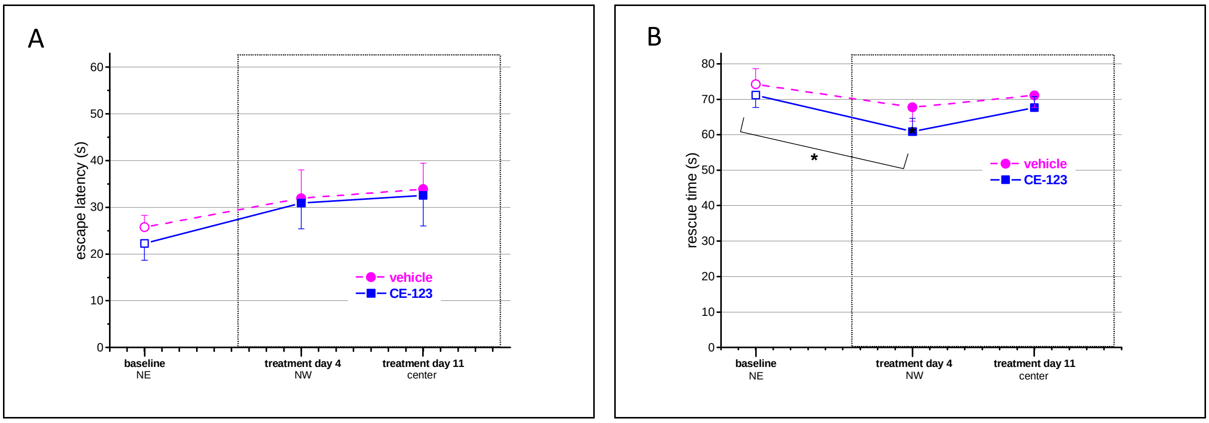

Figure S2. Performance in the Morris water-maze of animals repeatedly treated with S-CE-123 or vehicle. (For the study design *see* Methods and Materials.) (A) Escape latency purified from the trials where the animals had to be rescued. (B) Rescue time (time spent in water until rescued, data from 7 and 11 rats in the vehicle- and S-CE-123-treated groups,repsectively). Shown are mean ±SEM values. Scaling of X-axis reflects calendar days. Dotted rectangle signs the treatment period. *: p<0.05 significance of the difference from baseline (repeated measures ANOVA followed by Duncan test); NE, NW, and 'center' indicates the position of the escape platform

ANOVA table escape latency

Effect	Repeated Measures Analysis of Variance (nonrescueddata_MWM in ce123_MWM) Sigma-restricted parameterization Effective hypothesis decomposition; Std. Error of Estimate: 15,308				
	SS	Degr. of Freedom	MS	F	p
Intercept	70630,56	1	70630,56	301,3994	0,000000
Treatment	77,65	1	77,65	0,3314	0,570006
Error	5858,55	25	234,34		
R1	1284,29	2	642,14	1,5030	0,232336
R1*Treatment	23,53	2	11,76	0,0275	0,972854
Error	21361,83	50	427,24		

ANOVA table rescue time

Effect	Repeated Measures Analysis of Variance (rescue_time_MWM in ce123_MWM) Sigma-restricted parameterization Effective hypothesis decomposition; Std. Error of Estimate: 13,891				
	SS	Degr. of Freedom	MS	F	p
Intercept	242904,2	1	242904,2	1258,747	0,000000
Treatment	253,0	1	253,0	1,311	0,269018
Error	3087,6	16	193,0		
R1	609,2	2	304,6	3,495	0,042373
R1*Treatment	37,1	2	18,6	0,213	0,809274
Error	2788,9	32	87,2		

Duncan-test

Cell No.	Duncan test; variable DV_1 (rescue_time_MWM in ce123_MWM) Approximate Probabilities for Post Hoc Tests Error: Within MSE = 87,154, df = 32,000			
	R1	{1} 72,343	{2} 63,546	{3} 68,998
1	Lat (NE) D1 mean		0,010776	0,290611
2	Lat (NW) D2 mean2	0,010776		0,089499
3	Lat (centre) D3 mean3	0,290611	0,089499	

Cooperation task: Number of initiated trials

ANOVA table

Effect	Repeated Measures Analysis of Variance (ce123coop_stat in ce123_coop) Sigma-restricted parameterization Effective hypothesis decomposition; Std. Error of Estimate: 77,5015 Include condition: v14=1				
	SS	Degr. of Freedom	MS	F	p
Intercept	792793,5	1	792793,5	131,9896	0,000000
Treatment	12015,4	1	12015,4	2,0004	0,171255
Error	132142,6	22	6006,5		
DAYS	270,2	3	90,1	0,1897	0,903062
DAYS*Treatment	4552,8	3	1517,6	3,1966	0,029017
Error	31333,5	66	474,8		

Duncan-test

Cell No.	Duncan test; variable DV_1 (ce123coop_stat in ce123_coop) Approximate Probabilities for Post Hoc Tests Error: Between; Within; Pooled MSE = 1857,7, df = 33,051 Include condition: v14=1									
	Treatment	DAYS	{1} 87,250	{2} 82,000	{3} 76,000	{4} 73,500	{5} 89,917	{6} 98,333	{7} 110,17	{8} 109,83
1	cont	Coop_ITI_day0		0,5572	0,2385	0,1644	0,8806	0,5583	0,2548	0,2510
2	cont	Coop_ITI_day1	0,5572		0,5025	0,3734	0,6757	0,4057	0,1691	0,1673
3	cont	Coop_ITI_day2	0,2385	0,5025		0,7797	0,4784	0,2670	0,1001	0,0992
4	cont	Coop_ITI_day3	0,1644	0,3734	0,7797		0,4140	0,2248	0,0808	0,0806
5	ce123	Coop_ITI_day0	0,8806	0,6757	0,4784	0,4140		0,3476	0,0396	0,0368
6	ce123	Coop_ITI_day1	0,5583	0,4057	0,2670	0,2248	0,3476		0,2150	0,2007
7	ce123	Coop_ITI_day2	0,2548	0,1691	0,1001	0,0808	0,0396	0,2150		0,9703
8	ce123	Coop_ITI_day3	0,2510	0,1673	0,0992	0,0806	0,0368	0,2007	0,9703	

Cooperation task: % successful trials

ANOVA table

Effect	Repeated Measures Analysis of Variance (ce123coop_stat in ce123_coop) Sigma-restricted parameterization Effective hypothesis decomposition; Std. Error of Estimate: 39,1796 Include condition: v14=1				
	SS	Degr. of Freedom	MS	F	p
Intercept	72895,77	1	72895,77	47,48792	0,000001
Treatment	2482,36	1	2482,36	1,61713	0,216772
Error	33770,84	22	1535,04		
DAYS	141,54	3	47,18	0,46224	0,709602
DAYS*Treatment	1059,04	3	353,01	3,45855	0,021216
Error	6736,62	66	102,07		

Duncan-test

Cell No.	Duncan test; variable DV_1 (ce123coop_stat in ce123_coop) Approximate Probabilities for Post Hoc Tests Error: Between; Within; Pooled MSE = 460,31, df = 31,238 Include condition: v14=1									
	Treatment	DAYS	{1} 26,501	{2} 22,044	{3} 17,634	{4} 23,705	{5} 26,483	{6} 32,393	{7} 36,170	{8} 35,517
1	cont	Coop_successful ITI0		0,3325	0,0578	0,5280	0,9985	0,5062	0,3231	0,3399
2	cont	Coop_successful ITI1	0,3325		0,2890	0,6885	0,6375	0,3013	0,1710	0,1863
3	cont	Coop_successful ITI2	0,0578	0,2890		0,1699	0,3656	0,1481	0,0765	0,0843
4	cont	Coop_successful ITI3	0,5280	0,6885	0,1699		0,7533	0,3744	0,2210	0,2386
5	ce123	Coop_successful ITI0	0,9985	0,6375	0,3656	0,7533		0,1816	0,0378	0,0478
6	ce123	Coop_successful ITI1	0,5062	0,3013	0,1481	0,3744	0,1816		0,3938	0,4516
7	ce123	Coop_successful ITI2	0,3231	0,1710	0,0765	0,2210	0,0378	0,3938		0,8749
8	ce123	Coop_successful ITI3	0,3399	0,1863	0,0843	0,2386	0,0478	0,4516	0,8749	

Cooperation task: number of rewards

ANOVA table

Effect	Repeated Measures Analysis of Variance (ce123coop_stat in ce123_coop) Sigma-restricted parameterization Effective hypothesis decomposition; Std. Error of Estimate: 54,5875 Include condition: v14=1				
	SS	Degr. of Freedom	MS	F	p
Intercept	108407,0	1	108407,0	36,38075	0,000005
Treatment	5612,0	1	5612,0	1,88337	0,183783
Error	65555,4	22	2979,8		
DAYS	690,8	3	230,3	0,85986	0,466422
DAYS*Treatment	3090,5	3	1030,2	3,84684	0,013373
Error	17674,3	66	267,8		

Duncan-test

Cell No.	Duncan test; variable DV_1 (ce123coop_stat in ce123_coop) Approximate Probabilities for Post Hoc Tests Error: Between; Within; Pooled MSE = 945,79, df = 34,623 Include condition: v14=1									
	Treatment	DAYS	{1} 32,167	{2} 23,500	{3} 20,167	{4} 28,000	{5} 29,000	{6} 40,667	{7} 47,833	{8} 47,500
1	cont	Coop_rew0		0,2441	0,1143	0,5616	0,8025	0,5030	0,2641	0,2577
2	cont	Coop_rew1	0,2441		0,6196	0,5031	0,6838	0,2320	0,1005	0,1010
3	cont	Coop_rew2	0,1143	0,6196		0,2746	0,5283	0,1607	0,0648	0,0654
4	cont	Coop_rew3	0,5616	0,5031	0,2746		0,9371	0,3662	0,1746	0,1748
5	ce123	Coop_rew0	0,8024	0,6838	0,5283	0,9371		0,1033	0,0123	0,0121
6	ce123	Coop_rew1	0,5030	0,2320	0,1607	0,3662	0,1033		0,3176	0,3102
7	ce123	Coop_rew2	0,2641	0,1005	0,0648	0,1746	0,0123	0,3176		0,9605
8	ce123	Coop_rew3	0,2577	0,1010	0,0654	0,1748	0,0121	0,3102	0,9605	

Nosepoke – leverpress discrimination: Number of initiated trials

ANOVA table

Effect	Repeated Measures Analysis of Variance (ce123np_leverpress_stat in ce123_np_lev_press) Sigma-restricted parameterization Effective hypothesis decomposition; Std. Error of Estimate: 93,6587				
	SS	Degr. of Freedom	MS	F	p
Intercept	1821618	1	1821618	207,6639	0,000000
Treatment	67615	1	67615	7,7081	0,010263
Error	219299	25	8772		
TIME	46735	3	15578	21,7705	0,000000
TIME*Treatment	22937	3	7646	10,6846	0,000006
Error	53667	75	716		

Duncan-test

Cell No.	Duncan test; variable DV_1 (ce123np_leverpress_stat in ce123_np_lev_press) Approximate Probabilities for Post Hoc Tests Error: Between; Within; Pooled MSE = 2729,7, df = 37,975									
	Treatment	TIME	{1} 94,923	{2} 105,00	{3} 104,38	{4} 115,38	{5} 98,286	{6} 152,93	{7} 182,21	{8} 186,57
1	cont	ITI-1		0,380636	0,392186	0,079780	0,868281	0,013533	0,000322	0,000195
2	cont	ITI-2	0,380636		0,952616	0,316833	0,756417	0,028872	0,000861	0,000557
3	cont	ITI-3	0,392186	0,952616		0,319630	0,763610	0,031633	0,000937	0,000585
4	cont	ITI-4	0,079780	0,316833	0,319630		0,446455	0,069936	0,002816	0,001946
5	ce123	ITI-1	0,868281	0,756417	0,763610	0,446455		0,000033	0,000025	0,000021
6	ce123	ITI-2	0,013533	0,028872	0,031633	0,069936	0,000033		0,005893	0,002374
7	ce123	ITI-3	0,000322	0,000861	0,000937	0,002816	0,000025	0,005893		0,673709
8	ce123	ITI-4	0,000195	0,000557	0,000585	0,001946	0,000021	0,002374	0,673709	

Nosepoke – leverpress discrimination: % successful trials

ANOVA table

Effect	Repeated Measures Analysis of Variance (ce123np_leverpress_stat in ce123_np_lev_press) Sigma-restricted parameterization Effective hypothesis decomposition; Std. Error of Estimate: 69,5677				
	SS	Degr. of Freedom	MS	F	p
Intercept	294572,3	1	294572,3	60,86621	0,000000
Treatment	29783,1	1	29783,1	6,15395	0,020198
Error	120991,7	25	4839,7		
TIME	13835,4	3	4611,8	17,45815	0,000000
TIME*Treatment	4532,1	3	1510,7	5,71882	0,001402
Error	19812,2	75	264,2		

Duncan-test

Cell No.	Duncan test; variable DV_1 (ce123np_leverpress_stat in ce123_np_lev_press) Approximate Probabilities for Post Hoc Tests Error: Between; Within; Pooled MSE = 1408,0, df = 33,558									
	Treatment	TIME	{1} 29,126	{2} 32,397	{3} 32,651	{4} 48,401	{5} 41,386	{6} 69,063	{7} 80,432	{8} 84,637
1	cont	succITI1		0,602982	0,600247	0,005958	0,447210	0,018485	0,003050	0,001566
2	cont	succITI2	0,602982		0,967921	0,020263	0,563282	0,027740	0,004894	0,002586
3	cont	succITI3	0,600247	0,967921		0,018658	0,549783	0,025684	0,004543	0,002439
4	cont	succITI4	0,005958	0,020263	0,018658		0,630661	0,162200	0,042419	0,026386
5	ce123	succITI1	0,447210	0,563282	0,549783	0,630661		0,000099	0,000050	0,000031
6	ce123	succITI2	0,018485	0,027740	0,025684	0,162200	0,000099		0,073458	0,020018
7	ce123	succITI3	0,003050	0,004894	0,004543	0,042419	0,000050	0,073458		0,503954
8	ce123	succITI4	0,001566	0,002586	0,002439	0,026386	0,000031	0,020018	0,503954	

Nosepoke – leverpress discrimination: number of rewards

ANOVA table

Effect	Repeated Measures Analysis of Variance (ce123np_leverpress_stat in ce123_np_lev_press) Sigma-restricted parameterization Effective hypothesis decomposition; Std. Error of Estimate: 136,8853				
	SS	Degr. of Freedom	MS	F	p
Intercept	906117,1	1	906117,1	48,35827	0,000000
Treatment	133545,7	1	133545,7	7,12716	0,013148
Error	468439,5	25	18737,6		
TIME	91069,5	3	30356,5	23,94552	0,000000
TIME*Treatment	40591,4	3	13530,5	10,67296	0,000006
Error	95079,9	75	1267,7		

Duncan-test

Cell No.	Duncan test; variable DV_1 (ce123np_leverpress_stat in ce123_np_lev_press) Approximate Probabilities for Post Hoc Tests Error: Between; Within; Pooled MSE = 5635,2, df = 35,688									
	Treatment	TIME	{1} 41,769	{2} 54,962	{3} 54,462	{4} 74,692	{5} 49,714	{6} 123,75	{7} 162,86	{8} 171,07
1	cont	reward1		0,388557	0,388478	0,033246	0,785190	0,015408	0,000525	0,000266
2	cont	reward2	0,388557		0,971121	0,154483	0,866208	0,029426	0,001215	0,000661
3	cont	reward3	0,388478	0,971121		0,168530	0,870612	0,033140	0,001369	0,000718
4	cont	reward4	0,033246	0,154483	0,168530		0,438901	0,098597	0,005914	0,003492
5	ce123	reward1	0,785190	0,866208	0,870612	0,438901		0,000032	0,000025	0,000021
6	ce123	reward2	0,015408	0,029426	0,033140	0,098597	0,000032		0,005745	0,001356
7	ce123	reward3	0,000525	0,001215	0,001369	0,005914	0,000025	0,005745		0,551112
8	ce123	reward4	0,000266	0,000661	0,000718	0,003492	0,000021	0,001356	0,551112	

Table S1. List of variables included in the the multivariate statistical analysis

abbreviation	description	vehicle mean± SEM	S-CE-123 mean± SEM	Category
**Motor activity assay (treatment day 15)**				
hor.act.	horizontal motor activity	618.5 ±42.35	775.7±45.87	motivational
**5-choice serial reaction time test (treatment days 11-12)**				
5-CSRTT IT	number of trials initiated	37.6 ±9.81	59.8 ±10.14	motivational
5-CSRTT reward	number of pellets obtained	9.7 ±4.14	13.9 ±3.44	success
5-CSRTT correct%	% correct (rewarded) trials	15.9 ±5.10	20.7 ±4.38	efficacy
5-CSRTT omission%	% trials omitted (without a response)	58.1 ±8.61	42.4 ±8.80	motivational
5-CSRTT premature%	% trials with premature response (not rewarded)	7.0 ±2.11	13.9 ±3.11	
**cooperation task (treatment day 10)**				
coop IT	number of trials initiated	73.5 ±15.77	109.8 ±10.05	motivational
coop reward	number of pellets obtained	28.0 ±8.90	47.5 ±12.25	success
coop IT%	% successful (rewarded) trials	23.7 ±6.34	35.5 ±7.77	efficacy
**nosepoke-leverpress discrimination (treatment day 15)**				
NPLP IT	number of trials initiated	115.4 ±15.18	186.6 ±15.25	motivational
NPLP reward	number of pellets obtained	74.7 ±21.95	171.1 ±20.08	success
NPLP IT%	% successful (rewarded) trials	48.4 ±11.61	84.6 ±7.44	efficacy
**Morris water-maze (treatment days 11-12)**				
MWM lat	average escape latency	45.0 ±6.58	46.2 ±7.56	efficacy
MWM no. escapes	number of trials when the platform was found in both sessions	5.15 ±0.53	4.5 ±0.53	success
**Pot jumping (treatment day 14)**				
PJ ld	longest distance jumped over	23.7 ±0.84	24.9 ±0.43	success
PJ-#jumps	number of all jumps in the session	8.7 ±1.16	10.6 ±1.15	motivational
PJ-ld eff	minimum number of jumps required to reach the farthest pot / number of jumps done until reaching the farthest pot	58.4 ±7.56	64.1 ±6.67	efficacy

Table S2. Multivariate ANOVA results (Wilks λ) of the behavioral assays. The first raw of the table contains the multivariate ANOVA results including all the 17 variables. Second to fourth raws: separately conducted multivariate ANOVA results on the 3 groups of parameters including (left side columns) or excluding (right side columns) variables of the NP-LP test.

Vaiables	Wilk's λ	F (df_{effect}, df_{error})	p value	Wilk's λ	F (df_{effect}, df_{error})	p value
	all tests			excluding nosepoke-leverpress discr.		
all	0,2713	1,4218 (17,9)	0,3017			
motivational PJ-#jumps, NPLP-ITI, coop-ITI, 5CSRTT-ITI, 5CSRTTomiss%, hor.act.	0.4592	3.9253 (6,20)	0.0094	0.5703	3.1645 (5,21)	0.0277
success NPLPrew, cooprew, 5CSRTTrew, MWM#esc, PJ-ld	0.5315	3.7014 (5,21)	0.0147	0.7289	2.0452 (4,22)	0.1229
efficacy NPLP-ITI%, 5CSRTTcorr%, coop-ITI%, MWMlat, PJ-ldeff	0.5865	2.9609 (5,21)	0.0355	0.7438	1.8946 (4,22)	0.1472

References:

1. Ernyey AJ, Bögi E, Kassai F, Plangár I, Gyertyán I (2019a): Translational difficulties in querying rats on 'orientation'. Biomed Res Int 2019:6149023.
2. Kozma K, Kassai F, Ernyey AJ, Gyertyán I (2019): Establishment of a rodent cooperation assay as a model of social cognition.*J Pharmacol Toxicol Meth* 97:44-51.
3. Ernyei AJ, Pereira Grohmann T, Kozma K, Kouhnavardi S, Kassai F, Gyertyán I (2019b): Following of aging process in a new motor skill learning model, "pot jumping" in rats. *Geroscience* 41:309-319.

Section 2: Plasma and brain levels of S-CE-123

LC-MS

For liquid chromatography (LC) an UltiMate 3000 RSLC-series system (Dionex; Thermo Fisher Scientific, Inc., Gemering, Germany) coupled with a maXis HD ESI-Qq-TOF mass spectrometer (Bruker Corporation, Bremen, Germany) was used. The separation was conducted with a Kinetex Phenyl-Hexyl 2.6 µm 100 Å reversed phase LC column (50x2.1 mm I.D, Phenomenex, Inc., Torrance, CA, United States) preceded by a suitable guard column. The settings of the ESI ion source were: Capillary voltage 3.5 kV; nebulizer 0.8 bar N_2; dry gas flow rate 7.0 L/min N_2; and dry temperature 200 ºC. Mass spectra were recorded in full-scan positive mode in the range of *m/z* 50-2500. Data were analyzed using Compass DataAnalysis 4.2 and QuantAnalysis 2.2 (Bruker Corporation). LC analyses were performed with a gradient elution of acetonitrile (ACN) (Merck KGaA, Darmstadt, Germany) (solvent B) and water (Merck KGaA, Darmstadt, Germany) containing 0.1 % formic acid (FA) (98 %, Carl Roth GmbH + Co. KG, Karlsruhe, Germany) (solvent A). The following solvent gradient was applied: 5 % solvent B at 0 min, 30 % solvent B at 5 min, 45 % solvent B at 10 min, 70 % solvent B at 12 min, a washing phase at 97 % solvent B from 13 to 14.5 min and column re-equilibration with 5 % solvent B from 14.5 to 15.5 min. The measurements were performed at a flow rate of 600 µL/min at an oven temperature of 40 ºC. Injection volume for all samples was 20 µL. The HRMS *m/z* values and LC retention times (RT) of *S*-CE-123 and *R*-modafinil are given in Table S3. All UHPLC-HRMS measurements were achieved with an error $\Delta < 10$ ppm.

Table S3. UHPLC-HRMS: Overview of calculated *m/z* values of modafinil and CE-123 positively charged with H^+ and Na^+ and their respective retention times.

Test item	Sum Formula	RT* (min)	*m/z* $[M+H]^+$	*m/z* $[M+Na]^+$
R-modafinil	$C_{15}H_{15}NO_2S$	4.5	274.0878	296.0716
S-CE-123	$C_{17}H_{15}NOS_2$	6.2	314.0644	336.0487

*retention time

For all plasma samples an aliquot of 100 µL treated plasma was spiked with 100 µL ACN containing *R*-modafinil (10000 ng/mL in ACN), as internal standard (IS). Samples were centrifuged for 10 minutes at 4500*g* at 20 °C. The supernatant was mixed 1:2 (v/v) with water, filtrated (Micropur, PTFE, 15 mm, 0.20 µm, PP-casing, Altmann Analytik, München, Germany) and aliquoted into 200 µL vials. All rat plasma samples were prepared in triplicates and measured by LC-HRMS in triplicates. All rat brain tissue samples were weighted and homogenized in homogenization solution containing *R*-modafinil (10000 ng/mL in ACN) (1W:2V) as IS using Precellys Evolution homogenizer (Bertin Technologies SAS, France) and centrifuged for 10 minutes at 3000*g* at 20 °C. The supernatant was diluted 1:10 (v/v) with 0.1 % FA in water. SPE clean-up was performed using Oasis PRiME HLB µElution 96-well plate 3 mg (Waters Corporation, Austria). The SPE cartridges were preconditioned and equilibrated with 1 x 200 µL ACN and 0.1% FA in water each. The samples were loaded on the cartridges and washed with 2 x 200 µL 0.1% FA in water and 1 x 200 µL ACN/0.1% FA in water 10:90 (v/v). Analytes were eluted from SPE cartridge into collection plate with 2 x 25 µL ACN/0.1% FA in water 70:30 (v/v) and diluted with 50 µL of 0.1% FA in water. All brain tissue samples were measured *via* LC-HRMS in triplicates.

The samples were evaluated using a calibration curve for both matrices. Standards were prepared following the same sample preparation procedure as previously described but

including a certain amount of *S*-CE-123 dissolved in ACN. All spiked plasma and brain tissue standards were prepared in duplicates and measured as duplicate each.

<u>Method Validation</u>

The in-house validation was conducted according to the guidelines of the ICH (4) and the USFDA (5) and results are reported in Table S4. The assessment of linearity in an initial analyte concentration range of 650-8700 ng/mL in plasma and 325-5200 ng/g in brain tissue samples was successful with a correlation coefficient (R^2) ranging from 0.9965 to 0.9973. The minimum calibration range necessary for drug substance assays (80 to 120 percent of test concentration) was achieved for both matrices (4). Accuracy was estimated using control plasma and brain tissue samples at concentrations within the validation range, prepared in duplicates. The acceptance criteria for accuracy of ± 15 % nominal concentration was achieved (5). Following the guidelines, a sufficient level of intra- and inter-day precision was accomplished (5) (Table S4). Furthermore, autosampler stability studies for plasma samples were conducted and after three days in stored autosampler all samples achieved the respective acceptance criteria (5) with relative error ranging from 7.2 to 12.1%. Applying the Signal-to-Noise Approach for *R*-modafinil a LLOD of 5 ng/mL and for *S*-CE-123 a LLOQ of 6 ng/mL and a LLOD of 2 ng/mL were determined (4).

Table S4. Method validation parameters including concentration range, correlation coefficient (R^2), slope and intercept of calibration curves, accuracy range expressed as relative error (RE), intra precision range (IAP) and inter precision range (IEP) for both matrices.

Matrix	Conc. range	R^2	Slope	Intercept	RE [%]	IAP [%]	IEP [%]
Plasma	650 – 8700 ng/mL	0.9965	2.7942	0.0365	-8.1 – 6.2	1.3 – 2.2	1.0 – 2.3
Brain	325 – 5200 ng/g	0.9973	8.0819	0.0277	-6.3 – 9.7	4.8 – 5.8	-

Plasma and brain level of S-CE-123

The concentrations of *S*-CE-123 in rat plasma and brain tissue samples were calculated according to the calibration curves (Table S4). The results are listed in Table S5 and Table S6, respectively. In total, 8 plasma samples were tested for their *S*-CE-123 concentration (9 technical replicates for each sample) and 6 rat brain tissue samples (3 technical replicates for each sample). S-CE-123 concentrations were in the range from 1220 to 4080 ng/mL *S*-CE-123 in rat plasma with CV between 2.4 to 4.3% and from 1338 to 2931 ng/g *S*-CE-123 in rat brain tissue with CV between 1.3 to 7.9%. The mean concentration of all rat plasma samples was 3100 ng/mL with an overall CV of 32% (n=72) and of all brain tissue samples 1800 ng/g with CV of 31% (n=17).

Table S5. Plasma concentrations of S-CE-123 in individual animals (mean from 9 technical replicates).

Rat Number	Concentration of *S*-CE-123 [ng/mL]	CV [%]
**51**	4080	2.7
**52**	2240	3.6
**53**	2170	4.3
**54**	3630	3.7
**57**	3910	3.0
**58**	1220	3.4
**65**	3680	2.5
**66**	3850	2.4
**overall**	3100	32

CV: coefficient of variation

Table S6. Brain concentrations of S-CE-123 in individual animals (mean from 3 technical replicates).

Rat Number	Concentration of *S*-CE-123 [ng/mL]	CV [%]
**51**[a]	1720	2.0
**52**	1490	1.3
**53**	1340	4.5
**54**	1690	3.7
**57**	2930	7.9
**65**	1600	4.2
**overall**	1800	31

CV: coefficient of variation; a: mean from 2 technical replication

References:

4. ICH Harmonised Tripartite Guideline: Validation of analytical procedures: Text and Methodology Q2(R1) (1994) https://www.ich.org/fileadmin/Public_Web_Site/ICH_Products/Guidelines/Quality/Q2_R1/Step4/Q2_R1__Guideline.pdf (accessed 2018.09.18)
5. USFDA: Bioanalytical Method Validation: Guidance for Industry (2018) https://www.fda.gov/downloads/drugs/guidances/ucm070107.Pdf (accessed 2018.09.18)

Section 3: Label-free quantification

Protein sample preparation

All homogenization and centrifugation steps were carried out on ice and at 4°C. Brain tissues were homogenized in an ice-cold homogenization buffer (10mM HEPES, pH 7.5, 300 mM sucrose, 1× Protease Inhibitor Cocktail (PIC, Roche Molecular Biochemicals)) using a Dounce homogenizer; the homogenate was centrifuged at 1000x g for 10 min to remove cell debris and nuclei and the supernatant was collected. The pellet was resuspended again in the homogenization buffer and centrifuged at 1000x g for 10 min. The pooled supernatants were then centrifuged at 15000x g for 30 min to obtain the total membrane fraction enriched in synaptosomes and mitochondria. The resulting pellets were washed with 10 mM HEPES, pH 7.5, PIC and solubilized in 50 mM TEAB buffer (Sigma-Aldrich), 7 M urea, 2 M thiourea, 4% CHAPS, 100 mM DTT and PIC. The protein concentration was determined by Pierce™ 660nm Protein Assay (Thermo Scientific).

LC-MS/MS

Protein samples were digested 18 h with trypsin (Promega) using filter-aided sample preparation (FASP) (6) with 70 µg of protein per one reaction. Tryptic peptides were desalted using reversed-phase C18 stage tips (7) and reconstituted in 40 µL of 100mM TEAB (Sigma-Aldrich). The actual amount of peptides was determined by Pierce™ Quantitative Fluorometric Peptide Assay (Thermo Scientific). A volume corresponding to 6 µg of peptides was transferred from each sample into separate vial, dried at 30°C (Speed-vac, Eppendorf) and reconstituted in 17 µl of 5% formic acid. The peptides (5 µl injection volume) were separated by LC using the following gradient of solvent A (2% acetonitrile, 0.1% TFA in water) and solvent B (80% acetonitrile in water) [0-7.2 min 5% B; 7.2-230 min 5-32% B; 230-250 min 32-50% B; 250-255 min 90% B; 255-260 min 5% B]. MS analysis was performed by the Thermo Scientific™ Q Exactive™ Plus Orbitrap mass spectrometer (Thermo Scientific) in positive ion mode with the following settings: full-scan MS in the range of m/z 380–1800 at the resolution of 70 000 (at m/z 200). MS/MS scans were acquired at the resolution of 17 500 (m/z 200) through HCD fragmentation of 20 most intense ions at 27% normalized collision energy with a fixed mass of 100 m/z. A raw data were analysed by MaxQuant 1.6.17.0 using Andromeda searching engine and LFQ algorithm (8).

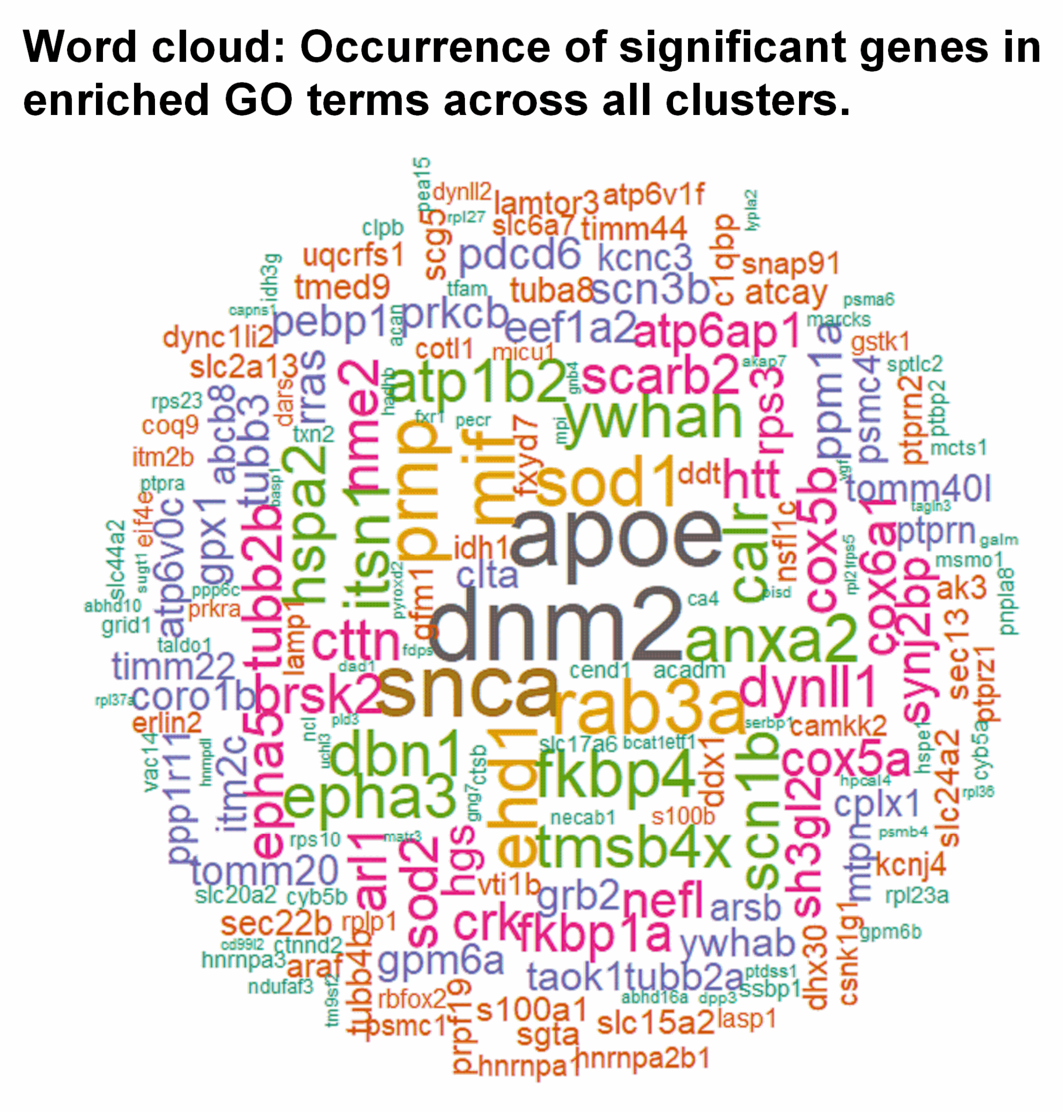

Figure S3. The occurrence of significant genes in enriched GO terms across all clusters is depicted in the size of the letters of each gene name. For building the word cloud the R-package “wordcloud” (9) was used.

References:

6. Wiśniewski JR, Zougman A, Mann MJ (2009): Combination of FASP and StageTip-based fractionation allows in-depth analysis of the hippocampal membrane proteome. *Proteome Res* 8:5674-5678. doi:10.1021/pr900748n
7. Rappsilber J, Mann M, Ishihama Y (2007): Protocol for micro-purification, enrichment, pre-fractionation and storage of peptides for proteomics using StageTips. Nat Protoc 2:1896-1906. doi:10.1038/nprot.2007.261.
8. Cox J, Mann M (2008): MaxQuant enables high peptide identification rates, individualized p.p.b.-range mass accuracies and proteome-wide protein quantification. Nat Biotechnol. 26: 1367-72.
9. Fellows I (2018): wordcloud: Word Clouds. R package version 2.6. https://CRAN.R-project.org/package=wordcloud
